# Neuronal Cascades Shape Whole-Brain Functional Dynamics at Rest

**DOI:** 10.1523/ENEURO.0283-21.2021

**Published:** 2021-10-20

**Authors:** Giovanni Rabuffo, Jan Fousek, Christophe Bernard, Viktor Jirsa

**Affiliations:** Aix Marseille University, INSERM, INS, Institut de Neurosciences des Systèmes, 13005 Marseille, France

**Keywords:** EEG/fMRI, network modeling, neuronal cascades, resting state

## Abstract

At rest, mammalian brains display remarkable spatiotemporal complexity, evolving through recurrent functional connectivity (*FC*) states on a slow timescale of the order of tens of seconds. While the phenomenology of the resting state dynamics is valuable in distinguishing healthy and pathologic brains, little is known about its underlying mechanisms. Here, we identify neuronal cascades as a potential mechanism. Using full-brain network modeling, we show that neuronal populations, coupled via a detailed structural connectome, give rise to large-scale cascades of firing rate fluctuations evolving at the same time scale of resting-state networks (*RSNs*). The ignition and subsequent propagation of cascades depend on the brain state and connectivity of each region. The largest cascades produce bursts of blood oxygen level-dependent (BOLD) co-fluctuations at pairs of regions across the brain, which shape the simulated *RSN* dynamics. We experimentally confirm these theoretical predictions. We demonstrate the existence and stability of intermittent epochs of *FC* comprising BOLD co-activation (CA) bursts in mice and human functional magnetic resonance imaging (fMRI). We then provide evidence for the existence and leading role of the neuronal cascades in humans with simultaneous EEG/fMRI recordings. These results show that neuronal cascades are a major determinant of spontaneous fluctuations in brain dynamics at rest.

## Visual Abstract

**Figure:**
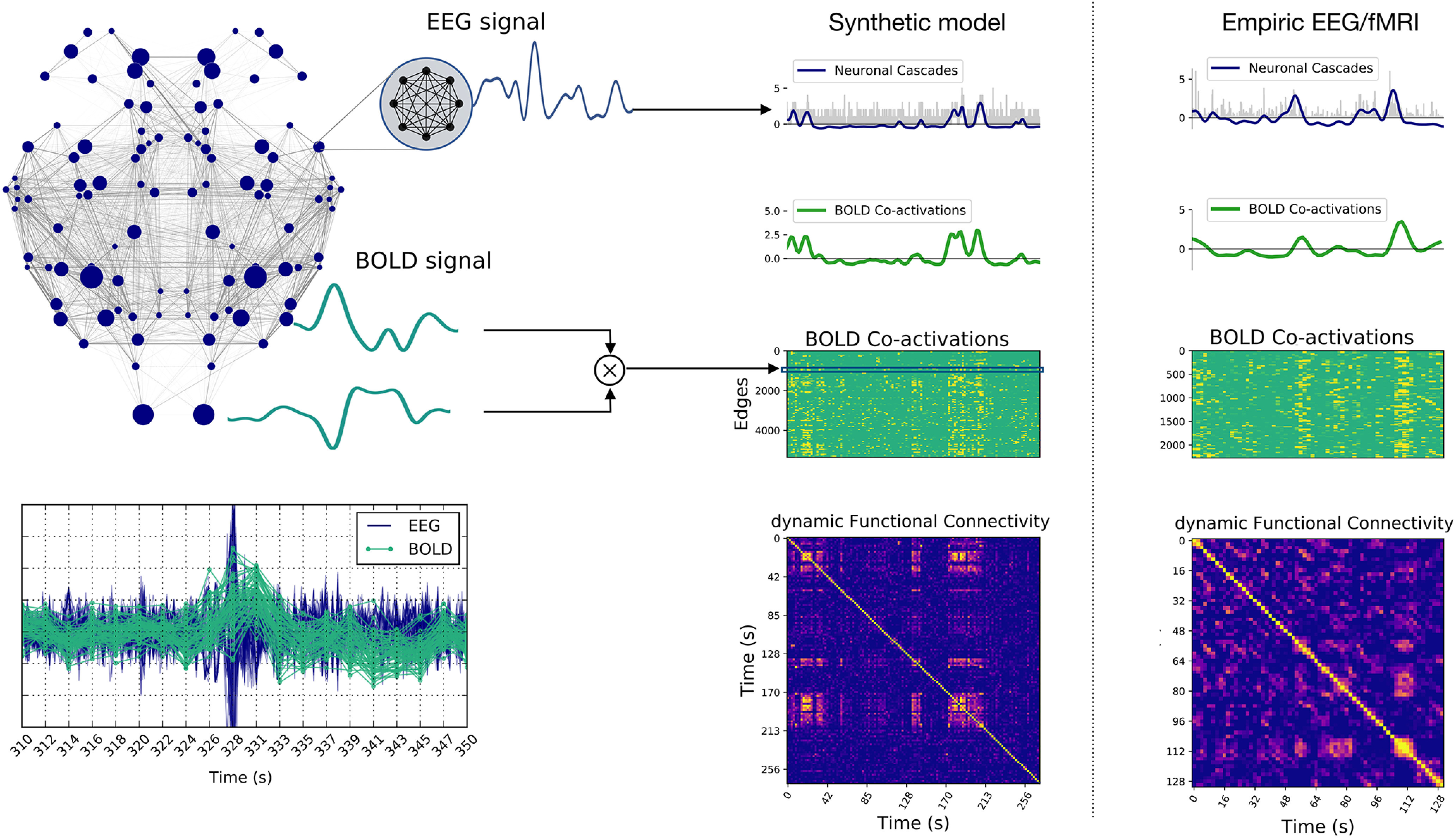

## Significance Statement

Functional connectivity (*FC*) and its dynamics are widely used as a proxy of brain function and dysfunction. Their neuronal underpinnings remain unclear. Using connectome-based modeling, we link the fast temporal microscopic neuronal scale to the slow emergent whole-brain dynamics. We show that cascades of neuronal activations spontaneously propagate in resting state-like conditions. The largest neuronal cascades result in the co-fluctuation of blood oxygen level-dependent (BOLD) signals at pairs of brain regions, which in turn translate to stable brain states. Thus, we provide a theoretical framework for the emergence and the dynamics of resting-state networks (*RSNs*). We verify these predictions in empirical mouse functional magnetic resonance imaging (fMRI) and human EEG/fMRI datasets measured in resting states conditions. Our work sheds light on the multiscale mechanisms of brain function.

## Introduction

**Figure 1.**
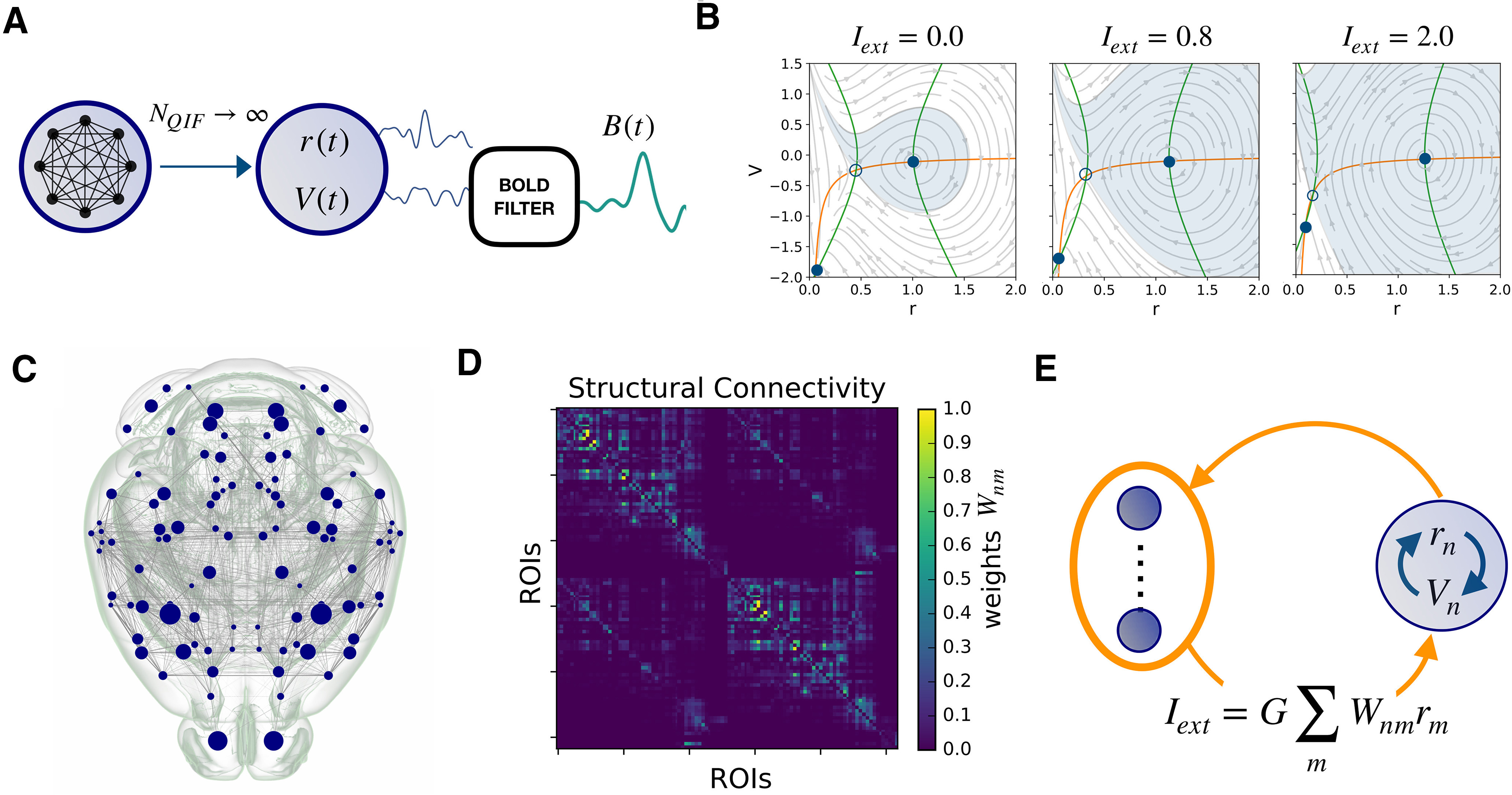
Connectome based modeling. ***A***, The mean firing rate *r* and membrane potential *V* variables of the NMM are derived as the limit of infinite all-to-all coupled QIF neurons. Applying the Balloon–Windkessel model to *V _n_*(*t*) we obtain the simulated BOLD signal *B_n_*(*t*) at node *n*. ***B***, The phase plane of each decoupled node (*I_ext_* = 0) has a “down” stable fixed point and an “up” stable focus (full dots). These points are defined at the intersection of the nullclines $\dot{r}=0$ (orange line) and $\dot{V}=0$ (green line) where the dynamics freezes. The empty circle marks an unstable fixed point. As the external current *I_ext_* is increased, the phase plane of the neural mass changes (see equations in Materials and Methods). In particular, the basin of attraction of the up state gradually becomes larger than that of the down state, while the fixed points move farther apart. ***C***, ***D***, The mouse connectome and structural connectivity *W_nm_* were imported from the tracer experiments of the Allen Institute. The 104 cortical ROIs (corresponding to network nodes) are specified in Table 1. ***E***, When the regions are coupled in a brain network, each node *n* receives an input current *I_ext_* which is the sum of the other nodes’ firing rates, weighted by the structural connectivity. According to panel ***B***, this input provokes a distortion of the local phase plane at node *n*.

At rest, functional magnetic resonance imaging (fMRI) reveals the existence of periods during which blood oxygen level-dependent (BOLD) activity is highly correlated between specific brain regions, known as resting-state networks (*RSNs*). *RSNs* are consistently observed across several mammalian species including humans (Fox and Raichle, 2007; Power et al., 2011) and non-human (Vincent et al., 2007) primates, as well as in rats (Upadhyay et al., 2011; Lu et al., 2012) and mice (Sforazzini et al., 2014; Stafford et al., 2014; Gozzi and Schwarz, 2016; Grandjean et al., 2020). Functional connectivity (*FC*) can be used to characterize these strongly correlated functional communities of brain network nodes. A growing body of research on mammalian species emphasizes the dynamic nature of *RSNs*, showing that large-scale *FC* patterns switch between stable and unstable epochs at an infraslow pace (<0.1 Hz; Tagliazucchi et al., 2012b; Allen et al., 2014; Qin et al., 2015; Hindriks et al., 2016; Grandjean et al., 2017; Preti and Van De Ville, 2017; Gonzalez-Castillo and Bandettini, 2018). Dynamic *FC* (*dFC*; Hutchison et al., 2013) measures *FC* fluctuations and provides a marker of healthy, aging, and diseased brains (Damaraju et al., 2014; Rashid et al., 2014; Su et al., 2016; Du et al., 2017; Preti et al., 2017; Battaglia et al., 2020). Our current understanding of these phenomena rests on computational studies, performed at the whole-brain level, suggesting that the *RSN* dynamics is an emergent property of the network (Ponce-Alvarez et al., 2015), which operates near criticality (Ghosh et al., 2008; Deco and Jirsa, 2012; Jirsa et al., 2017). Nevertheless, the range of possible mechanistic underpinnings remains vast and requires further narrowing down. Here, we address this issue and investigate *in silico* the potential neuronal mechanisms giving rise to whole-brain network dynamics, generate predictions and test them *in vivo*. We use The Virtual Brain (TVB), a neuroinformatics and simulation platform, which allows connectome-based whole-brain modeling of multiple species including humans (Sanz-Leon et al., 2015) and mice (Melozzi et al., 2017). TVB includes a fixed number of network nodes (at least one per brain region) and the physical connections that link them, such as white matter tracts defined by diffusion tensor imaging or axon projections obtained with virus injections (Sanz Leon et al., 2013). A large number of neural mass models (NMMs) are readily available in TVB, which produce activity of neuronal populations and map on a range of brain imaging modalities including BOLD fMRI, EEG, and MEG signals. We here adapt a novel NMM, which has been previously derived as the exact limit of an infinite number of all-to-all coupled quadratic integrate-and-fire (QIF) neurons (Montbrió et al., 2015; Fig. 1*A*). This analytic step allows to derive the average firing rate and membrane potential of a mesoscopic neuronal population, thus providing suitable neural mass variables while keeping track of the internal spiking neural network organization, which is important to infer potential mechanisms at the neuronal scale. Using this NMM, we report a mechanism by which spontaneous local re-organizations of regional spiking neural networks, can trigger global infra-slow fluctuations, which we name neuronal cascades. The largest neuronal cascades give rise to bursts of simulated BOLD co-activations (BOLD-CAs) at pairs of brain regions across the brain, which in turn account for stable long-lasting *FC* states and their dynamics. We verify experimentally the link between BOLD-CA and *dFC* in mouse fMRI and human EEG-fMRI. On the simultaneous human EEG acquisition, we discover the presence of neuronal cascades and we demonstrate their role in driving the switching behavior of *FC* states. These findings provide the first evidence of a multiscale mechanism underlying *RSN* dynamics which can be robustly assessed in non-invasive brain imaging signals.

## Materials and Methods

### Empirical mouse connectome

The connectome used for the simulations was extracted using The Virtual Mouse Brain pipeline described previously (Melozzi et al., 2017), which processes the tracer experiments performed at the Allen Institute (Oh et al., 2014). There, adult male C57Bl/6J mice are injected in specific brain regions with a recombinant adeno-associated virus, which expresses the EGFP anterograde tracer. In each experiment, the tracer migration signal is detected by a serial two-photon tomography system. The anterograde tracing provides information about the axonal projections starting at the injected site. We define the injection density of the source region as the number of infected pixels normalized by the total number of pixels in the region. Similarly, the projection density is defined in every region as the number of pixels detected in the target region following an injection at the source, normalized by the total number of pixels belonging to the target region. The tracer-based connectome is built by averaging over injection experiments performed in the right brain areas and targeting regions in ipsilateral and contralateral hemispheres. Through the Allen Connectivity Builder interface in TVB we parceled the brain in 104 anatomic regions of interest (ROIs; Table 1). Then, we defined the connection strength between source region *n* and target region *m*, i.e., the structural edge *nm*, as the ratio between the projection density at *m* and the injection density at *n*. The tracer structural connectome *W*, with edges normalized between 0 and 1 is shown in Figure 1*D*.

**Table 1:** List of brain ROIs of the Allen Mouse Atlas considered in the simulations

ROI ID:	ROI name:	ROI ID:	ROI name:
0	Right primary motor area	52	Left primary motor area
1	Right secondary motor area	53	Left secondary motor area
2	Right primary somatosensory area, nose	54	Left primary somatosensory area, nose
3	Right primary somatosensory area, barrel field	55	Left primary somatosensory area, barrel field
4	Right primary somatosensory area, lower limb	56	Left primary somatosensory area, lower limb
5	Right primary somatosensory area, mouth	57	Left primary somatosensory area, mouth
6	Right primary somatosensory area, upper limb	58	Left primary somatosensory area, upper limb
7	Right supplemental somatosensory area	59	Left supplemental somatosensory area
8	Right gustatory areas	60	Left gustatory areas
9	Right visceral area	61	Left Visceral area
10	Right dorsal auditory area	62	Left dorsal auditory area
11	Right primary auditory area	63	Left primary auditory area
12	Right ventral auditory area	64	Left ventral auditory area
13	Right primary visual area	65	Left primary visual area
14	Right anterior cingulate area, dorsal part	66	Left anterior cingulate area, dorsal part
15	Right anterior cingulate area, ventral part	67	Left anterior cingulate area, ventral part
16	Right agranular insular area, dorsal part	68	Left agranular insular area, dorsal part
17	Right retrosplenial area, dorsal part	69	Left retrosplenial area, dorsal part
18	Right retrosplenial area, ventral part	70	Left retrosplenial area, ventral part
19	Right temporal association areas	71	Left temporal association areas
20	Right perirhinal area	72	Left perirhinal area
21	Right ectorhinal area	73	Left ectorhinal area
22	Right main olfactory bulb	74	Left main olfactory bulb
23	Right anterior olfactory nucleus	75	Left anterior olfactory nucleus
24	Right piriform area	76	Left piriform area
25	Right cortical amygdalar area, posterior part	77	Left cortical amygdalar area, posterior part
26	Right field CA1	78	Left field CA1
27	Right field CA3	79	Left field CA3
28	Right dentate gyrus	80	Left dentate gyrus
29	Right entorhinal area, lateral part	81	Left entorhinal area, lateral part
30	Right entorhinal area, medial part, dorsal zone	82	Left entorhinal area, medial part, dorsal zone
31	Right subiculum	83	Left subiculum
32	Right caudoputamen	84	Left caudoputamen
33	Right nucleus accumbens	85	Left nucleus accumbens
34	Right olfactory tubercle	86	Left olfactory tubercle
35	Right substantia innominata	87	Left substantia innominata
36	Right lateral hypothalamic area	88	Left lateral hypothalamic area
37	Right superior colliculus, sensory related	89	Left superior colliculus, sensory related
38	Right inferior colliculus	90	Left inferior colliculus
39	Right midbrain reticular nucleus	91	Left midbrain reticular nucleus
40	Right superior colliculus, motor related	92	Left superior colliculus, motor related
41	Right periaqueductal gray	93	Left periaqueductal gray
42	Right pontine reticular nucleus, caudal part	94	Left pontine reticular nucleus, caudal part
43	Right pontine reticular nucleus	95	Left pontine reticular nucleus
44	Right intermediate reticular nucleus	96	Left intermediate reticular nucleus
45	Right central lobule	97	Left central lobule
46	Right culmen	98	Left culmen
47	Right simple lobule	99	Left simple lobule
48	Right ansiform lobule	100	Left ansiform lobule
49	Right paramedian lobule	101	Left paramedian lobule
50	Right copula pyramidis	102	Left copula pyramidis
51	Right paraflocculus	103	Left paraflocculus

### Neural mass model

The dynamics of a brain ROIs (i.e., a node in the structural network) is described by a NMM derived analytically as the limit of infinitely all-to-all coupled *θ*-neuron phase oscillators, whose dynamics is equivalent to that of QIF neurons (Fig. 1*A* ; Montbrió et al., 2015). The *i*-th neuron is described by the equation

(1) $$\tau_{c}{\dot{V}}_{i}(t)=V_{i}^{2}(t)+\eta_{i}+J\bar{s}(t)+I(t)\text{ if }V_{i}\geq V_{p}\text{ then }V_{r}\leftarrow V_{i}.$$

As the membrane potential reaches a peak value *V_p_*, it resets to *V_r_*. The limit $V_{p}=-V_{r}\to \infty$ is considered. The parameter *η_i_* is the neuron excitability, and it enters as an heterogeneous current in the membrane potential. A time-dependent current *I*(*t*) homogeneously affects all the neurons in the neural mass. The synaptic activation *s*(*t*) of a single neuron respects the equation $Q\cdot s(t)=\sum_{k}\delta (t-t_{k})$, being $Q=(1-\tau \frac{\text{d}}{\text{d}t})$ and *t_k_* the arrival time of the presynaptic action potential. The neuronal coupling among *N_QIF_* neurons inside a neural mass is given by the average synaptic activation $\bar{s}(t)=\frac{1}{N_{QIF}}\sum_{j>1}^{N_{QIF}}s(t)$ scaled by the synaptic weight *J*. Assuming a Lorentzian distribution of the membrane potentials across the coupled neuronal population it is possible to perform the $N_{QIF}\to \infty$ limit exactly according to the Ott–Antonsen ansatz (Ott and Antonsen, 2008). Also, the heterogeneous currents are distributed according to a Lorentzian distribution with width at mid-height Δ and peak location *η*. After the limit we can describe the activity in a neural mass *n* in terms of the average membrane potential *V _n_*(*t*) and the average firing rate *r_n_*(*t*) (which corresponds to $\bar{s}(t)$ in the limit *τ* → 0). The dynamics associated with a local network node is then described by the following NMM equations:

(2) τc r˙n(t)=Δπ + 2rn(t)Vn(t),

(3) $$\tau_{c}{\dot{V}}_{n}(t)=V_{n}^{2}(t)+\eta +Jr_{n}(t)-\pi^{2}r_{n}^{2}(t)+I(t).$$

The synaptic weight *J *=* *14.5, the average neuronal excitability *η* = – 4.6, the spread of the heterogeneous noise distribution Δ = 0.7, and the characteristic time *τ_c_* = 1 are homogeneously tuned so that each decoupled node is in a bistable regime with a “down” fixed point and an “up” stable focus in the 2D phase space (Fig. 1*B*, *I_ext_* = 0). Differently from standard NMMs, the chosen model quantifies the internal amount of synchronization of the constituent neurons by preserving the analytic form of the Kuramoto parameter (Kuramoto, 2003)

(4) $$Z_{n}(t)=|\sum_{i}\exp(i\theta_{i})|=K_{n}(t)\exp(i\theta_{n}(t)),$$

during the thermodynamic limit. The phase-oscillator representation (i.e., in terms of *θ_i_*) of the QIF equations can be achieved from Equation 1 by a change of variables $V_{i}=\tan(\theta_{i}/2)$. *K_n_* and *θ_n_* are the average amount of synchronization and the average phase of the neurons in the node *n*. The real and imaginary components of *Z_n_* are connected to the average membrane potential *V_n_* and firing rate *r_n_* of the neural population *n* via the relation:

(5) $$Z_{n}=\frac{1-w_{n}^{*}}{1+w_{n}^{*}},\;w_{n}\equiv \pi r_{n}+iV_{n}.$$

### Connectome-based modeling

The local model is then coupled over an empirically extracted connectome (Fig. 1*C*,*D*). The coupling term enters as an additive current in the average membrane potential equations (see also Fig. 1*E*):

(6) $$I(t)=G\sum_{m\neq n}W_{nm}r_{m}(t-\tau_{nm}).$$

The global coupling parameter *G* sets the impact of the connectivity matrix *W_nm_* over the local dynamics. The time delay $\tau_{nm}=l_{nm}/v$ is given by the tract length between nodes *n* and *m* divided by the average conduction speed which we set to a realistic value *v *=* *4 *m*/*s*. Since the firing rate is by definition greater than or equal to zero, all the interactions are excitatory.

The numeric integration of the nodal equations over the network is performed using TVB open-source neuroinformatics platform (Sanz Leon et al., 2013). The solution of the coupled system consists of a neuroelectric raw dataset describing the evolution of the variables $\left(r_{n}(t),V_{n}(t)\right)$ in each region *n* of the connectome. These variables are our measure of microscopic activity. The sampling rate of these neuroelectric variables is set to 1000 Hz. The surrogate BOLD activity *B_n_*(*t*) in each region is derived by filtering the membrane potential with the Balloon–Windkessel model (Friston et al., 2000; default values implemented in TVB). We use a repetition time of 2 s so that the BOLD rate is 0.5 Hz.

Note that the conduction speed *v* is a function of the physical tract lengths *l_nm_* of the empirical connectome and of the resolution *Dt* of the simulated signal, which physical interpretation is arbitrary. We impose it to be *Dt *=* *1 ms. Accordingly, 2000 time steps correspond to 2 s* *=* *1 BOLD point. All the dimensional arguments treated in the text are based on this convention. In particular, we adopted a nondimensional formalism for the NMM Equation 2, where only the characteristic time constant *τ_c_* has the dimension of time.

### Mouse and human empiric datasets

The empirical mouse fMRI dataset was imported from the publicly available collection Grandjean (2020). For our analysis, we used the cohort of 20 control wild-type (resting) animals registered in the subdataset (Mandino, et al., 2019). The data were previously preprocessed according to a common pipeline (https://github.com/grandjeanlab/MouseMRIPrep), and registered on the Allen volumetric atlas. In our analysis, we considered the activity in those voxels corresponding to the imported Allen connectome (Table 1). The empiric dataset did not distinguish between specific parts (e.g., ventral and dorsal) of certain brain regions (Anterior cingulate, Retrosplenial and Entorhinal areas). A unique time series was associated with each of these pairs of regions.

The empirical human EEG/fMRI dataset was acquired and preprocessed at Charité-Universitätsmedizin Berlin (Schirner et al., 2018) and made available in online repository (https://osf.io/mndt8/; Schirner, 2018). In summary, from a larger cohort (49 subjects, 18–80 years), 15 youngest subjects (18–31 years) were selected based on the quality of the EEG recording after correction of the MR artifacts. For each subject, diffusion-weighted MRI, T1-weighted MRI, and EEG-fMRI in resting-state paradigm were obtained. The T1-weighted image was used to parcellate the cortical gray matter into 68 regions according to the Desikan–Killiany atlas (Desikan et al., 2006). This definition of regions was then used to estimate the structural connectivity from the dw-MRI data (Schirner et al., 2015), and to extract the regional average time series from the fMRI data. The EEG data (Easy-cap; 64 channels, MR compatible) was treated with a high pass filter at 1.0 Hz followed with MRI acquisition artifact removal using Analyser 2.0 (v2.0.2.5859, Brain Products). The resulting sensor-level time series was downsampled to 200 Hz and low pass filtered to 30 Hz before correction for physiological artifacts (ballistocardiogram, muscle activity). Next, EEG source imaging was performed to obtain projected activity on the cortical surface and averaged within the 68 regions of the Desikan–Killiany parcellation. See Schirner et al. (2018) for a detailed description of both fMRI and EEG processing. After the preprocessing, we made a quality check of every subject data and excluded BOLD points and associated EEG time windows [BOLD time repetition (TR) = 1.94 s = 388 EEG samples] which presented residual artifacts (at either EEG or fMRI level) based on the following criteria: if any of the six time series of the motion degrees of freedom (resulting from the FSL MCFLIRT head movement correction step) presented a peak; if the EEG window contained fluctuations simultaneously affecting most of the frequencies (time-frequency analysis); and if the EEG presented fluctuations above 7 SDs.

We finally selected 30 artifact-free trials of consecutive EEG/fMRI acquisition (minimum duration 2 min) across the cohort.

### Time-dependent *FC*

To quantify the temporal evolution of the brain *FC*, we have employed two approaches: windowed *dFC* (*dFC_w_*), and edge-*dFC* (*dFC_e_*). Let us denote by *B_n_*(*t*) the regional BOLD time-series for each node *n *=* *1…*N*. To compute the *dFC_w_* (Allen et al., 2014), we first obtain the series of *FC* matrices *FC*(*w*) at each sliding window *w *=* *1…*N_w_*, defined as the correlation matrices for the segments $B_{n}(t){|}_{t_{w}}^{t_{w}+\tau }$ (we fix the window size *τ* = 60 s and window step 2 s). Next, we correlate the vectorized upper triangular parts of the *FC*(*w*) matrices at different time windows to obtain the *dFC_w_* matrix:

(7) $$dFC_{w}(w_{i},w_{j})=corr\left[triu(FC(w_{i}),triu(FC(w_{j}))\right].$$

**Figure 2.**
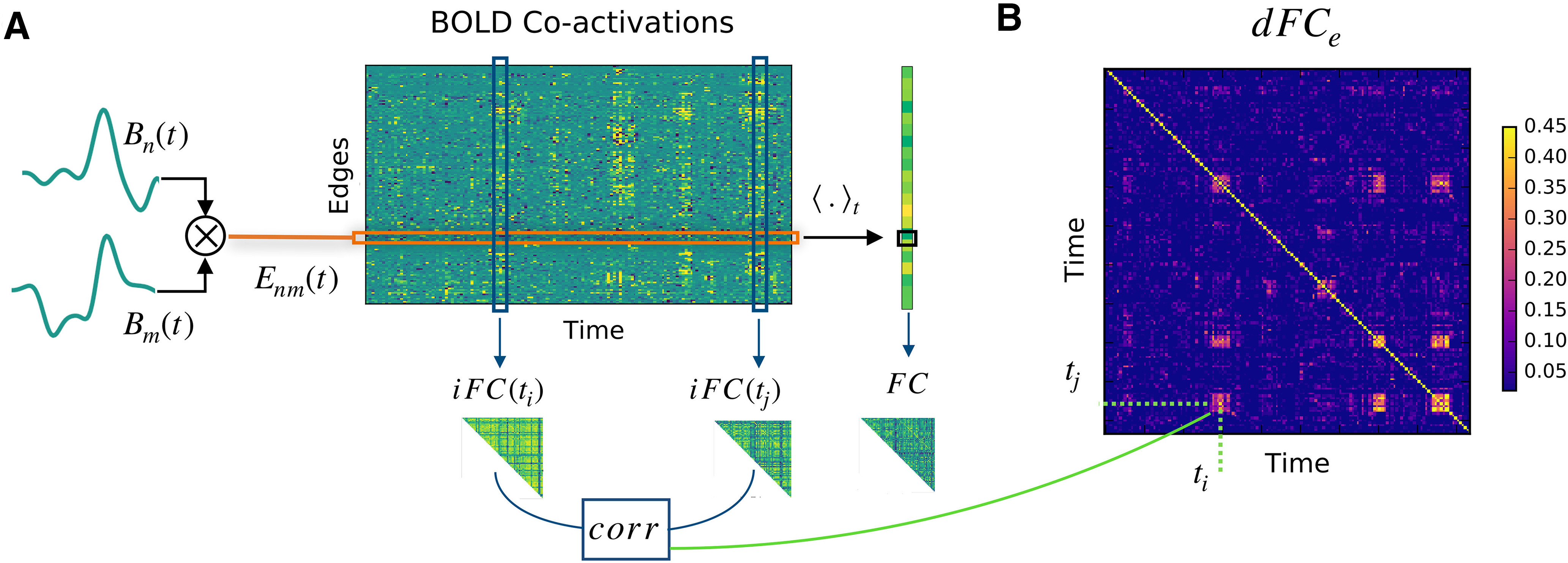
Two regimes of *dFC*. ***A***, Given two nodes *n* and *m* the edge CA signal *E_nm_*(*t*) (orange box) is defined as the product of the z-scored BOLD signal *B_n_*(*t*) and *B_m_*(*t*). Averaging the BOLD-CA matrix over time we obtain the Pearson correlation across each pair of brain regions *n* and *m* (in black box, right), defining the static *FC*. Each column of the BOLD-CA matrix represents an istantaneous realization of the *FC* (*iFC*). ***B***, The elements (*t_i_*, *t_j_*) of the *dFC_e_* matrix are defined as the Pearson correlation between *iFC*(*t_i_*) and *iFC*(*t_j_*). Note in panel ***A*** the presence of transient bouts of strong BOLD-CA (e.g., in the blue boxes). During these events, the *iFC* remains relatively correlated for few consecutive time points, which gives rise to diagonal (yellow) blocks in the *dFC_e_* matrix. The same CA burst (e.g., at *t_i_*) can re-occur in time after long periods of time (e.g., at *t_j_*), which gives rise to an off-diagonal *dFC_e_* block (e.g., at the crossing of the dashed lines in panel ***B***).

On the other hand, the computation of the *dFC_e_* starts with the z-scored BOLD time series *B_n_* (subtract the mean and divide by the SD). The edge time series is then computed as element wise multiplication along time for each pair of regions $E_{nm}(t)=B_{n}(t)\cdot B_{m}(t)$ for *n*,*m *=* *1…*N* (see Fig. 2*A*). Next, for each pair of time points *t*_1_, *t*_2_, we compute the *dFC*_e_ matrix elements (Fig. 2*B*) as the correlation:

(8) $$dFC_{e}(t_{i},t_{j})=corr(E_{nm}(t_{i}),E_{nm}(t_{j})).$$

A main difference among the *dFC* variants lies in the scope of z-scoring of the time series *B_n_*. In the case of *dFC_e_* the *z* score *z_n_* is computed from the whole time series, whereas in the *dFC_w_* the *z* score is performed within the Pearson correlation in each time window $\left[t_{w},t_{w}+\tau \right]$ separately.

### Surrogate BOLD models

In order to compare our results below (Results, Neuronal cascades subtend RSN dynamic) about the origin of the *FC* dynamics with the null hypotheses of a random evolution and of inter-regional stationarity of the *FC*, we build time-shuffled and phase-randomized surrogates of the *FC* dynamics, respectively. The time-shuffled surrogate is obtained by randomizing the order of the instantaneous *FC*s *iFC*(*t*), i.e., the columns of the edge CA time series *E_nm_*(*t*) (see Fig. 2*A*). According to Hindriks et al. (2016) the phase randomized surrogate is obtained by adding a uniformly distributed random phase to each frequency of the Fourier transformed signal, and subsequently retrieving the phase randomized signals by anti-Fourier transform. Importantly, the phase shift is different at every time point but can be applied uniformly to all the brain regions (cross-spectrum preserved) or separately in every region (cross-spectrum not preserved). Only in the first case the bursts of CA are not destroyed but shifted (Extended Data Fig. 9-1*A*) and the static *FC* is preserved. The coherent fluctuations around the stationary *FC*, however, are destroyed. For more details, see also Prichard and Theiler (1994).

## Results

We start by introducing the synthetic setup used to simulate the main feature of *RSN* dynamics, i.e., the intermittent epochs of stable *FC*. To achieve recurrent functional network activation we set the brain regions into a bistable regime, where the mean membrane potentials can exhibit a resting (down) and a depolarized (up) state (Hansen et al., 2015). When the regions are suitably coupled over a connectome, the local activity spontaneously fluctuates between these states, which promotes system metastability, i.e., the recurrent exploration of multiple network configurations (Deco et al., 2017; Beim Graben et al., 2019). In order to understand the mechanisms allowing the dynamic coordination of bistable neural masses into multistable network re-configurations, we looked in detail at the building blocks of our simulation.

### Rules of single and coupled nodes dynamics

Let us first consider the dynamics of a single isolated brain node (not connected to the network) where the local NMM parameters are tuned to ensure bistability. If there is no input current (*I_ext_* = 0), solving the model equations (see Materials and Methods) resolves the dynamics of the node in terms of the mean firing rate *r*(*t*) and mean membrane potential *V*(*t*). The motion trajectories are represented on the (*r*, *V*) phase plane (Fig. 1*B*, left panel).

If, at a given time, a node takes a given $\left(r^{*},V^{*}\right)$ value, the direction of the arrow at these coordinates indicates the direction of the flow, and where the node dynamics will end up: either falling in the down stable state where the neurons populating the NMM exhibit low firing rate and strong (negative) membrane potential; or spiraling in a damped oscillation into the up stable state, in which the neurons display high average firing rate and low average membrane potential. Thus, in the absence of noise, neuronal activity of the node would rapidly freeze into a stable up or down level of activity (Fig. 1*B*, full dots).

Introducing noise allows the stochastic exploration of the phase space. If the noise is sufficiently large, a random movement in the phase plane can provoke the sudden switch from up→down or down→up basins of attraction (Fig. 1*B*, light blue and white shades, respectively), therefore changing the *r* and *V* dynamic modality.

We now consider a non-null input current *I_ext_*, simulating a constant synaptic input. As *I_ext_* increases (Fig. 1*B*), the two branches of the *V*-nullcline ( $\dot{V}=0$ lines) move horizontally away from each other, while the *r*-nullcline ( $\dot{r}=0$ line) remains fixed. Correspondingly, the location of the stable points in the phase plane shifts (at $\dot{V}=\dot{r}=0$) and their basins of attraction change in size. The separatrix, i.e., the line separating the up and down basins of attraction, is therefore a dynamical element: its location depends on the amount of input that the node is receiving at a given time.

If *I_ext_* is small, the separatrix is close to the up state (Fig. 1*B*, left) and the crossing of the separatrix via random fluctuations is more probable in the up → down direction; the node explores only the down state. Conversely, if the separatrix is close to the down state when *I_ext_* is high (Fig. 1*B*, right), the node likely remains in the up state.

This property of the NMM is key to understand the system’s dynamic. At a given time *t* the inputs received by node *n* will shift the separatrix, and make it easier (or not) for the node to change its activity as a function of noise and of its current state [ $\left(r^{*},V^{*}\right)$ coordinates at time *t*].

Using this NMM, we simulate whole-brain dynamics in a mouse brain avatar (Melozzi et al., 2017, 2019), the detailed connectivity of which is imported from the tracer experiments of the Allen Institute and is parcellated into anatomic ROIs, associated with the network nodes (Fig. 1*C*; Oh et al., 2014). The communication between regions is weighted by the structural links *W_nm_* of the structural connectivity (Fig. 1*D*). Thus, the input *I_ext_* of a node represents the synaptic drive from the firing rates *r_m_* of all the *m* nodes that are connected to node *n* (Fig. 1*E*; see Materials and Methods). As an effect of the coupling, the (projected) phase plane (*r_n_*, *V _n_*) at node *n* can be thought of as a distorted version of the phase plane of a single isolated node, where the location of the separatrix depends on the structural role that region *n* plays in the network hierarchy. Less connected regions receive a weak input current *I_n_* so that the local phase plane is slightly distorted. Instead, regions with high centrality in the connectome, or that are part of strongly connected structural motifs, receive a strong input, which causes a greater distortion of the local phase plane, with consequences on their activity.

### CA bursts account for *RSN* dynamics

Once a working regime is chosen by the selection of global and local model parameters, the raw outcome of the simulation consists of a high time-resolution neuroelectric signal: the average firing rate *r* and membrane potential *V* for each node. As a convention, we refer to the average firing rate activity as the simulated EEG data. With a further processing step, we obtain a low time-resolution simulated BOLD activity by filtering the membrane potentials through the Balloon–Windkessel model (Friston et al., 2000; Fig. 1*A*, right).

In order to compare simulated and empirical whole-brain imaging data, we analyze the dynamic evolution of functional brain patterns with *dFC* measures (Majeed et al., 2011; Smith et al., 2012; Hutchison et al., 2013; Lindquist et al., 2014; Karahanoğlu and Van De Ville, 2015; Shine et al., 2015). The most diffuse definition of *dFC*, is based on a sliding window approach (Allen et al., 2014). In brief, inside each time window, a static *FC* is computed as the correlation matrix of the BOLD activities. Then, the entries of the *dFC_w_* matrix are defined as the correlation between the *FC*s at different windows (see Materials and Methods). In typical empiric datasets, *dFC_w_* matrices show non-trivial block structures; diagonal block structures represent epochs of stable *FC*, while the off-diagonal blocks mark the re-occurrence of correlated *FC* at distinct times. For our analysis, we introduce a *dFC* measure derived in an edge-centric approach, where the dynamics for the edge *E_nm_* is defined by the product of the z-scored BOLD activities *B_n_* and *B_m_* at nodes *n* and *m* (Faskowitz et al., 2020). The resulting edge CA time series (Fig. 2*A*, orange box) tracks the temporal unfold of correlations across node pairs.

In fact, averaging the edge CAs across time defines the Pearson correlation across each pair *n* and *m* (Fig. 2*A*, right, black box). The correlation for each couple of regions (i.e., each edge) defines the static *FC* (Fig. 2*A*, right). Thus, we can interpret the columns in Figure 2*A*, blue boxes, as instantaneous realizations of the *FC* (*iFC*) at different times *t*. The *dFC_e_* (Fig. 2*B*) is defined (without the use of sliding windows) by the Pearson correlation of the *iFC*s at each couple of times *t_i_* and *t_j_*. On the one hand, the window approach to *dFC* allows a more reliable measure of the correlations to the detriment of the resolution over the temporal structure. On the other hand, the edge approach is more sensitive to spurious correlations but, crucially for our following analysis, it maintains the full time resolution of the BOLD signals. Notably, the example Figure 2*A* extracted from a regime of interest, reveals that short duration bouts of strong BOLD-CAs (vertical stripes) spontaneously appear in association with a non-trivial *dFC_e_*. As a final remark, we also notice that the *dFC_e_* blocks (Fig. 2*B*) occur in coincidence with the strongest edge CAs, an observation that will have important consequences in our final analysis.

### Multiple pathways to simulate *RSN* dynamics

**Figure 3.**
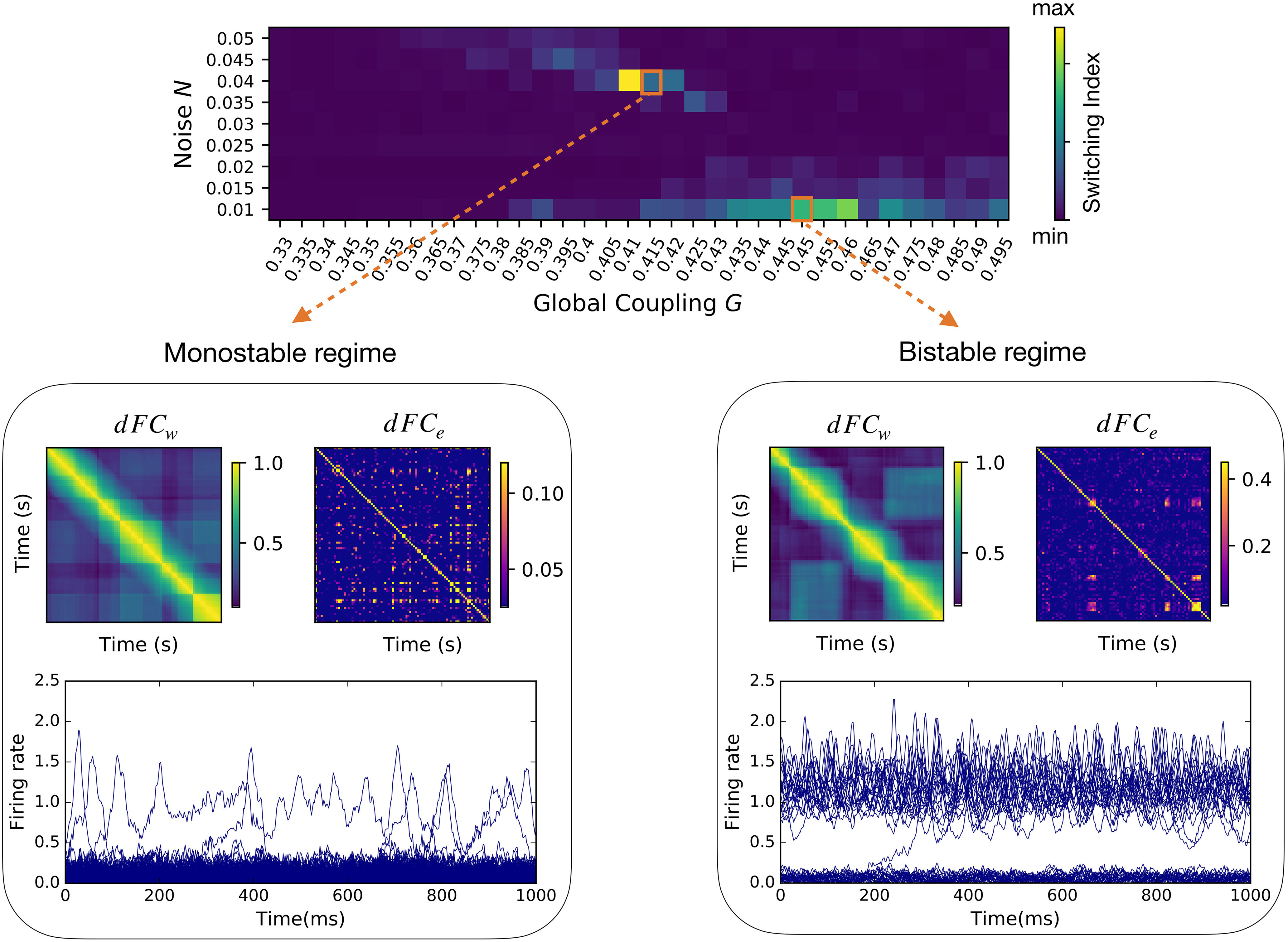
Two qualitatively distinct regimes of non-trivial functional dynamics. For every couple of global parameters (*G*, *N*) we calculated the *dFC* in a sliding window approach (*dFC_w_*; as in Materials and Methods) and in an edge-centric approach (*dFC_e_*; as in Fig. 2*A*,*B*). The “switching index” of each *dFC_w_* matrix was evaluated as the variance of the respective upper triangular elements. We find two regimes of activity, named monostable and bistable, where qualitatively distinct neuroelectric organizations give rise to large-scale functional dynamics characterized by a non-vanishing switching index. In both regimes, the *dFC_w_* and *dFC_e_* display off-diagonal blocks, demonstrating a correlation between the functional activity at distinct times. The low global coupling *G* in the monostable regime (bottom left) does not guarantee a strong communication between the brain network regions, which most of the time populate the low firing rate (“down”) state. A strong noise *N* pushes the brain regions in the high firing rate (“up”) state for short transients. A higher value of the global coupling in the bistable regime (bottom right) promotes a subgroup of regions in the high firing rate (up) state. Low levels of noise perturb the equilibrium of the system provoking localized switching in both up →down and down →up directions (e.g., at *t *=* *200 ms).

To search for regimes of dynamic resting-state activity, we explore the parameter space by varying the global synaptic coupling *G* and the noise *N*, representing the impact of the structure over the local dynamics, and the stochastic currents simulating a generic environmental noise (thermodynamic, chemical, synaptic…), respectively. Then, we look for regions of the parameter space where a non-trivial functional network dynamics emerge. We define the “switching index,” i.e., the variance across all elements of the upper triangular part of the *dFC_w_* matrix (Fig. 3) to quantify the temporal irregularity of functional activity (roughly speaking, the number of different *dFC_w_* block structures and how often they switch between one another). We identify two regimes giving rise to *RSN* dynamics, where the respective neuroelectric correlates are qualitatively different.

In the first case, a weak structural coupling (low *G*) is insufficient to promote any region into a high firing rate state, because the excitatory drive received by any node is relatively small. However, all regions can transiently reach a high firing rate state because the stochastic drive is strong. We refer to this as the monostable regime (Fig. 3, bottom left). In the second case, the stronger structural coupling pushes a subset of regions into a strongly active state leaving the remaining regions in the down state. A weak stochastic drive ensures stable dynamics with few regions jumping between the up and down states. We call this the bistable regime (Fig. 3, bottom right).

In conclusion, the balance between the local dynamics and the global connectivity (tuned by *G*), together with appropriate levels of perturbation of the system (*N*), allow the same NMM model to generate diverse large-scale organizations qualitatively similar to those measured empirically.

Before moving on, let us notice that the block structures in the *dFC_w_* (examples in Fig. 3, bottom panels) give the impression that whole-brain dynamics is organized by a sequence of transient but long-lasting stable periods of correlated activity. However, the analysis of the corresponding *dFC_e_* reveals that whole-brain dynamics is in fact characterized by much smaller periods of CAs. Since we want to compare resting-state dynamics to the underlying fast neuronal activity, we are interested in the highest temporal resolution available. Therefore, unless differently specified, in the following analysis we use *dFC_e_*. We also notice that when *dFC_w_* is used, as the window slides in time, it will capture the transient edge CA bursts as long as the latter is present in the window, giving rise to spurious long-lasting block structures (e.g., see the different block organization between *dFC_w_* and *dFC_e_* in Fig. 3, bottom right). Next, we analyze the neuronal mechanisms underlying resting-state functional dynamics generated by the model.

### A generative mechanism for slow cascades of neuronal activations

Functional dynamics and the underlying neuronal activity fundamentally differ in their intrinsic time scales. Neuronal fluctuations typically evolve at the milliseconds scale. *RSN* dynamics unfolds in the order of tens of seconds. In this section, studying the results of our simulation, we reveal a potential mechanism by which local neuronal perturbations can generate a slow cascade of activation across the brain network, thus approaching the temporal scale of *RSN* dynamics.

**Figure 4.**
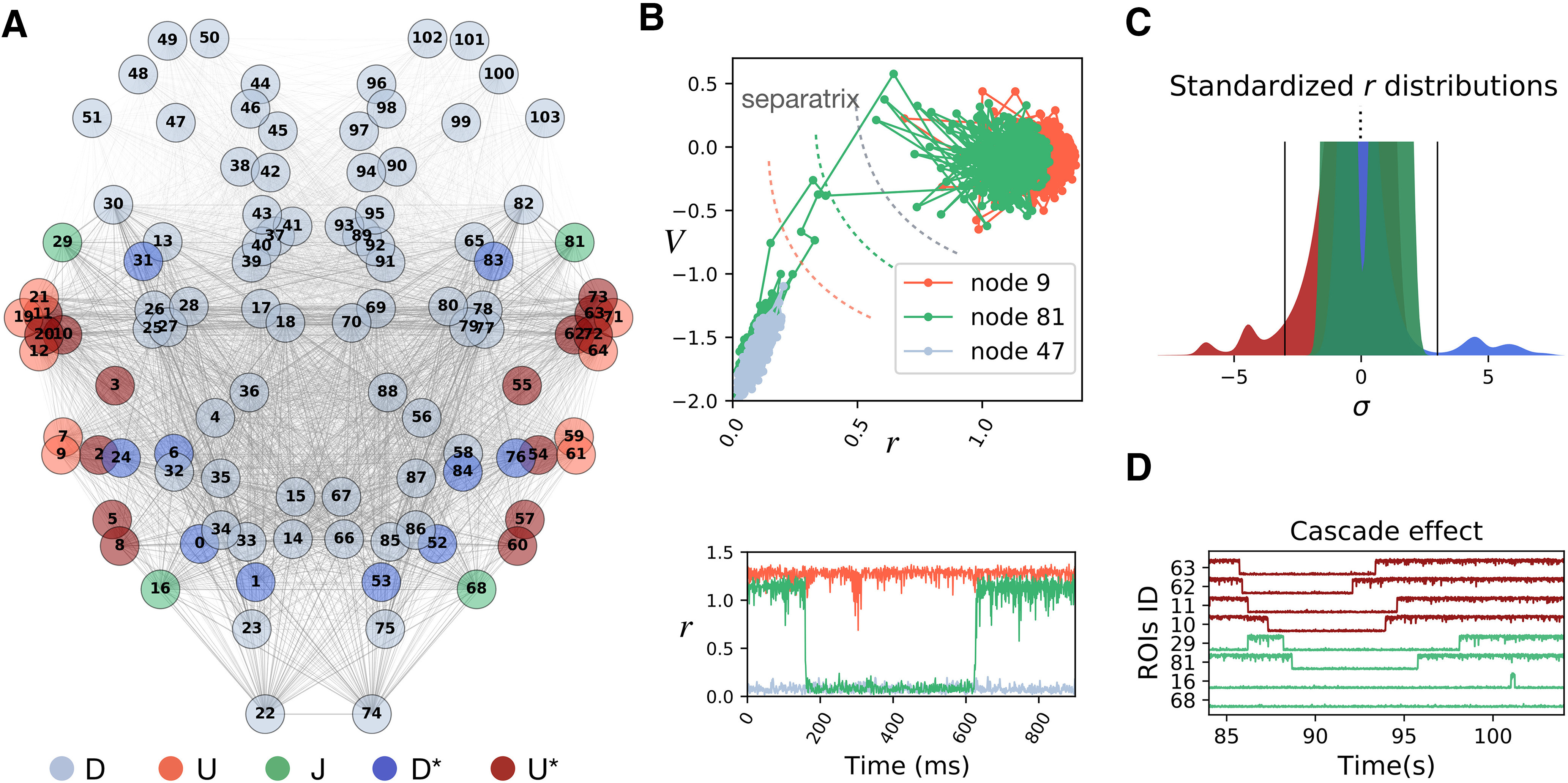
Mechanisms of cascade generation in the synthetic model. ***A***, Different regions have a different fate depending on their location in the connectome. We classified the regions in five classes (D, U, J, D*, U*) according to their activity. ***B***, Example exploration of the projected 2D phase space (top) and firing rates activity (bottom) of the “up-U” (light red), “down-D” (light blue), and “jumping-J” (green) regions. ***C***, Distribution of the standardized firing rates in different classes. Class (J) regions have two modes but never cross the ±3 σ threshold (black lines). Class (U*) (dark blue) and class (D*) (dark red) regions dwell most of the time in the up and down states, respectively. Only in important rare occasions the *-regions cross the threshold to jump on the other side, substantially deviating from their baseline activity. The leading role of the *-regions as compared with the other classes is shown using PCA in Extended Data Figure 4-1*A*,*B*. ***D***, Example of a cascade: when the (U*) node 63 jumps into the down state, it first drags down the node 62 (with which it shares the strongest structural link in the network). After them, other strongly connected nodes follow the trend.

To do so, we introduce a framework to interpret the simulated firing rate dynamics and its dependence on the structural connectome. We focus on the bistable regime where the low noise level allows a clearer visualization of the dynamic mechanisms in act. The hypotheses developed here will be later tested on the monostable regime as well as in empiric data. We identify five main categories of nodes based on their simulated firing rate activity:

- (D) down regions (Fig. 4*A*, light blue) display a low firing rate state throughout the duration of the simulation, as the up state is practically unreachable by noise-driven fluctuations. According to the section above, Rules of single and coupled nodes dynamics, the separatrix lies close to the up state (Fig. 4*B*, top). Their low firing rate regime (Fig. 4*B*, bottom) makes them poor communicators in the network hierarchy.
- (U) up regions (Fig. 4*A*, light red) always show a high firing rate (Fig. 4*B*, bottom), constantly providing inputs to their targets. The separatrix is close to the down state and is never reached (Fig. 4*B*, top).
- (J) jumping regions (Fig. 4*A*, green) undergo regular local transitions, dwelling for a relatively long time in both low-firing and high-firing rate states (Fig. 4*B*, bottom). In the projected phase space of these regions, the separatrix lies midway between the up and down states (Fig. 4*B*, top). Therefore, they have the same probability to jump from up to down and vice versa Note that the timing of the (J) jumps defines a new slow time scale for the system. The fact that these jumps occur on a regular basis during the simulation ensures that this time scale is ever-present in the large-scale network dynamics.
- (D*) down-up regions (Fig. 4*A*, dark blue in network plot) have a stable activity around the down state fixed point but, in rare occasions, manage to reach the up state stable focus for a certain transient time.
- (U*) up-down regions (Fig. 4*A*, dark red in network plot) have a stable activity around the up state but are occasionally driven into a short-lived excursion to the down state.

**Figure 5.**
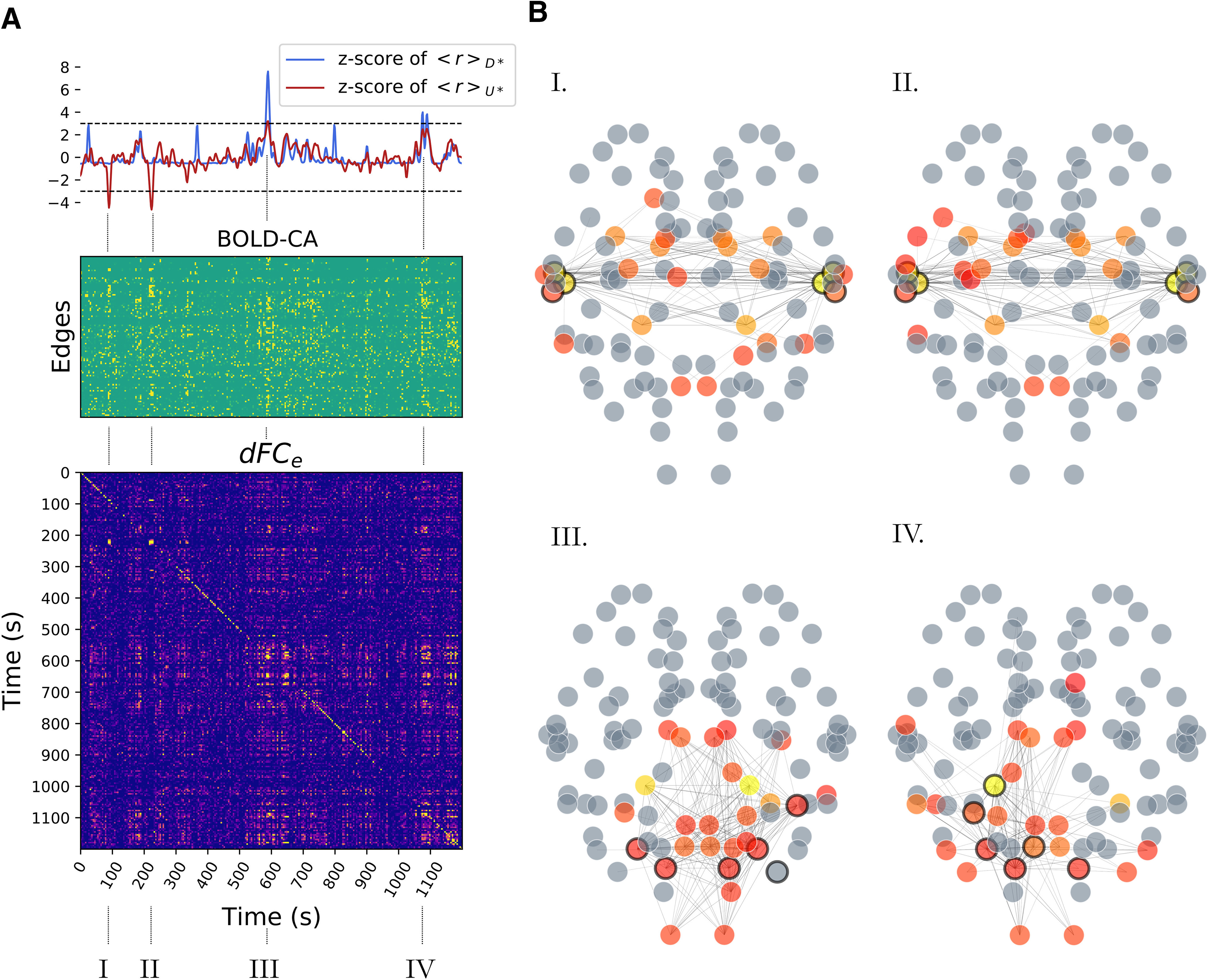
*RSN* formation. ***A***, top, The standardized firing rate activity in the (U*) and (D*) classes (class-specific average; dark red and dark blue, respectively) is characterized by peaks (the strongest are marked as I, II, III, IV) occurring in correspondence of cascades similar to Figure 4*D*. ***A***, middle, During a cascade, we also observe a peak of BOLD-CAs, appearing as vertical strips. Many, but not all, edges are recruited. ***A***, bottom, The blocks in the *dFC_e_* matrix appear in correspondence of CA events, showing that these bursts generate stable epochs of *FC* correlated in time. ***B***, In each selected epoch (I, II and III, IV), the large firing rate cascades trigger the jump of other nodes away from baseline activity (circled in black) and promote specific functional hubs at the BOLD level, represented by colored nodes in the network plots. A functional hub is defined by the components of the first leading eigenvector (linear combination of brain regions explaining most of the variance in the data; eigenvalue *λ* > 0.41) associated to the *iFC*s at times $t_{I},t_{II},t_{III}$, and *t_IV_*, respectively. The most representative hub regions are depicted in yellow. Gray regions have been excluded as they do not contribute substantially. Only the edges with the highest CAs are displayed. Importantly, CA events generated from neuronal cascades at specific sites support distinct functional networks which are not correlated among themselves (e.g., no off-diagonal *dFC_e_* block between I and III).

10.1523/ENEURO.0283-21.2021.f4-1 Extended Data Figure 4-1 ***A***, The first three principal components extracted from 1200 s of simulated firing rate activity in the bistable regime reveal a dominant contribution of the jumping regions (green ROIs corresponding to class J in Fig. 4*A*). ***B***, The analysis of the first principal component of the simulated firing rate extracted from nonoverlapping 2-s time windows (corresponding to 2000 firing rate time points) reveals the intermittent contribution of the regions from classes U* and D* (dark red and dark blue ROIs, respectively), which become active carriers of the system variance during times associated to the neuronal cascades (e.g., around *t *=* *600 s corresponding to Epoch III in Fig. 5*A*). Download Figure 4-1, EPS file.

The topographic organization of the firing rate classes shows the influence of the connectome in organizing whole-brain dynamics (Fig. 4*A*, regions labels in Table 1). Nodes receive different inputs as a function of the location in the connectome, which grants a different jumping probability and therefore a different role in network dynamics. A stochastic jump is a functionally important event for the network, as it corresponds to a sudden firing rate increase or decrease, which greatly influences the activity of downstream nodes. Thus, since the nodes of class (J) can jump regularly, we expect them to have a major role on whole-brain dynamics. A principal component analysis (PCA) of the regions’ firing rates shows that nodes (J) are the main contributors to the first three principal components, explaining most of the system variance (explained variance ratio >0.59; see Extended Data Fig. 4-1*A*).

Another key element of the simulated network dynamics is the occurrence of rare events, identified by large deviations, e.g., above 3 SDs σ away from baseline firing rate (Fig. 4*C*, black lines). Given their regular jumping, the activity of regions (J) never grows above 3 σ (Fig. 4*C* , bimodal distribution of standardized firing rate in green). The same holds for (D) and (U) regions. In contrast, we find that the regions in classes (D*) and (U*), occasionally grow above 3 σ during their rare jumps across the separatrix (Fig. 4*C*, dark blue and dark red distribution, respectively). Our simulation shows that these rare events can trigger cascades of transitions across the separatrix of a subset of regions (e.g., Fig. 4*D*). The propagation of a cascade depends on the structural location of the source node. Let us consider the case of a node with jumping potential, which is also a hub for the network (i.e., with high centrality; van den Heuvel and Sporns, 2013). Its local reshaping will have a wide influence throughout the network. However, the hubness of a jumping node does not guarantee the crossing of the separatrix by its target regions. If the hub is connected to a very stable subset of regions, following a jump of the hub, most of the separatrix lines in the targets will be slightly shifted, but not enough to allow any crossing. In contrast, if there exists a subset of strongly connected nodes with jumping potential, the jump of one will have strong effects within such subnetwork. In order to illustrate this point, let us consider that a group of nodes from class (U*), have very strong links binding them. Then, the occasional jump down of one of these nodes will strongly shift the separatrix of the other (U*) nodes toward the up stable focus, increasing their probability of jumping down. Then, many (U*) nodes will be dragged down one after the other, facilitating the next jumps, producing a cascade effect (as in Fig. 4*D*). During this event, the (J) nodes get also involved, interrupting for some time their regular control over the network. A windowed analysis of the first principal component of the firing rate activity shows that the nodes in the *-classes are driving the system during these rare events (Extended Data Fig. 4-1*B*).

The cascade effects quickly drag the system away from its standard state, establishing short epochs of increased deviations from baseline activity (the average firing rate in classes D* and/or U* deviates from baseline; Fig. 5*A*, top). In our simulation we mark four of these epochs (I, II, III, IV; Fig. 5*A*, bottom; Fig. 4*D* refers to Epoch I) which are separated by long periods of standard activity.

Summarizing, we have described how the jumps of the firing rate at local sites are extremely relevant for the unfolding of the neuronal activity throughout the brain, as they provoke the largest perturbations in the system. If jumps happen with a certain regularity, they will generate a slow time scale in the system. This slow rhythm in our simulations can be traced back to a small subset of regions (J) which transit between the up and the down states. These regions lead the baseline evolution of the entire system, acting as homeostatic agents which keep the system dynamics stable. On rare but important occasions this control weakens, which allows the propagation of a large cascade of activity. Such cascade effect takes place when a sudden local transition brings a typically stable region [(U*) or (D*)] away from baseline activity and induces other regions to reorganize. The cascade goes on until the noise and the structural pressure take the system back to its normal evolution, e.g., through the normalizing action of the jumping regions (J). We then explore whether such rare deviations from baseline activity account for resting-state brain dynamics.

### Neuronal cascades activate distinct RSN

The previous analysis focused on the simulated (high time resolution) neuronal activity. We now look at the simulated (low time resolution) BOLD, which signals are characterized by collective CA (Fig. 5*A*, middle). Interestingly, these events are aligned to the large deviations from baseline of the firing rate activity of the (U*) class or the (D*) regions (Fig. 5*A*, top, Segments I, II and III, IV, respectively). When looking at the corresponding *dFC_e_* matrix (Fig. 5*A*, bottom), we also note that the most stable *dFC_e_* blocks correspond to the largest BOLD-CA events. This observation is not a triviality, since a higher CA does not imply a stronger correlation. The presence of off-diagonal blocks in the *dFC_e_* shows that CA patterns are correlated when generated either by the (U*) or in the (D*) regions. In this example, the CA patterns generated by the jumps of distinct classes are not correlated. Each CA pattern defines the upper triangular part of an *iFC* (as in Fig. 2*A*). Figure 5*B* displays, for each selected epoch, the edges with the highest CA values. The functional hubs, a subset of regions with a central role in the functional dynamics, are defined in each epoch by the *iFC*’s leading eigenvector (Melozzi et al., 2017), and represented as different colors in the brain network plots (from red to yellow in a scale of importance). We highlight with a black rim the nodes whose firing rate deviated from baseline activity above a threshold (fixed at 3 σ). Notice that while the cascade generates at certain network locations (black rims), the dynamics of the RSN establishes a new emergent phenomenon at the large scale (colored nodes). The same perturbation can re-occur spontaneously eliciting similar functional networks correlated at distinct times, as it is marked by the off-diagonal blocks of the *dFC_e_* (Network Plots I, II or III, IV). Summarizing, neuronal perturbations starting at different locations in the connectome elicit different BOLD-CA events, which result in distinct functional networks.

### Neuronal cascades and BOLD-CAs in empiric data

**Figure 6.**
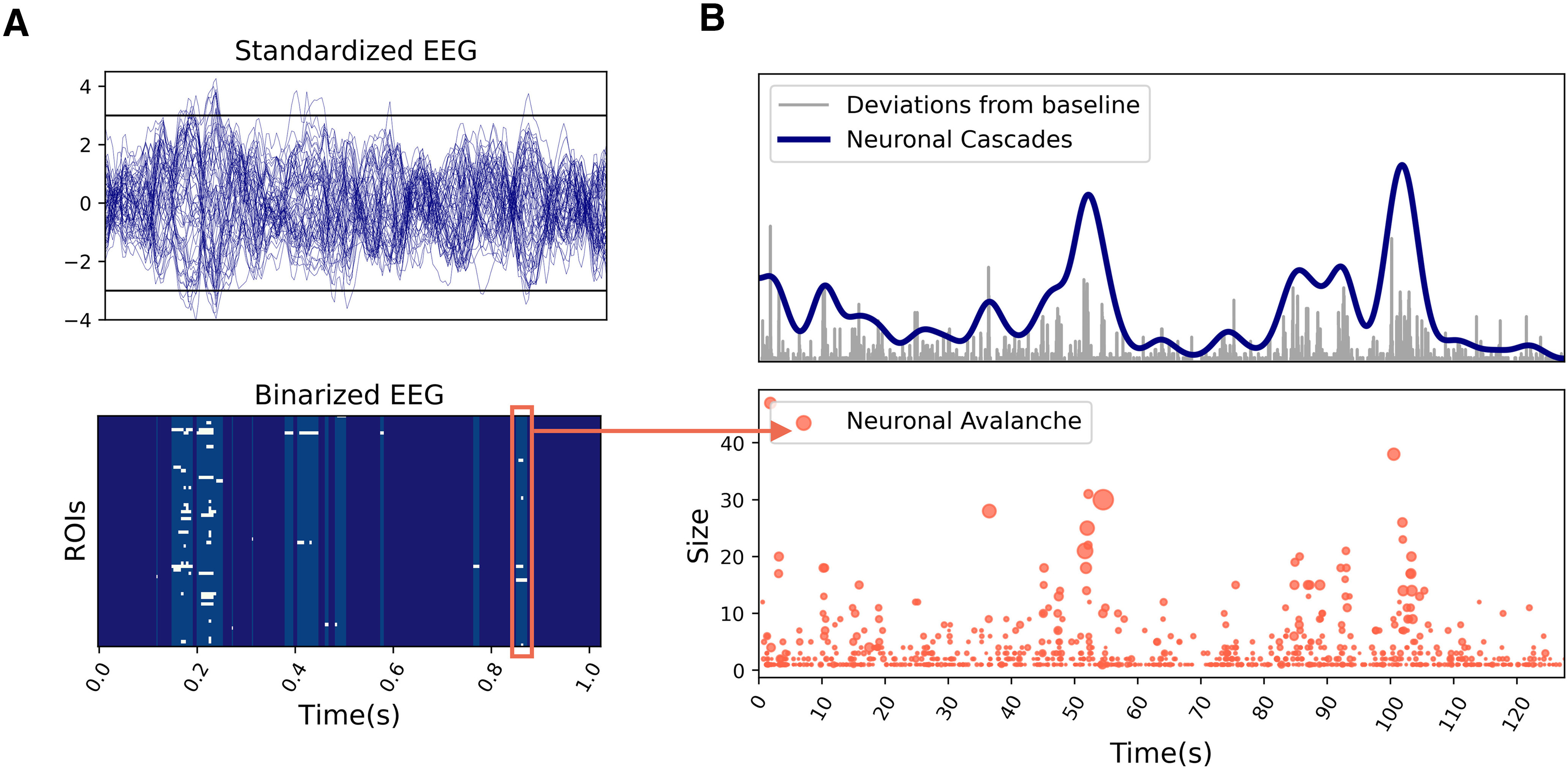
Neuronal cascades and neuronal avalanches. ***A***, Standardized EEG activity extracted from a resting-state human EEG/fMRI dataset (top). The activity is binarized assigning a unitary/null value every time the activity in a region is above/below a certain threshold (e.g., ±3 σ; black lines). The obtained binary raster plot (bottom) is characterized by intermittent epochs of deviations from baseline activity. Neuronal avalanches are defined as consecutive deviations from baseline activity (e.g., red box). ***B***, We extract the global magnitude of the deviations from baseline (top, gray signal) by summing the binary EEG raster plot over the ROIs. This signal is convoluted with a Gaussian kernel [width = 1 BOLD TR] and downsampled to obtain the same resolution of the BOLD activity, which defines the neuronal cascades signal (blue). Neuronal cascades can be thought of as clustering of high magnitude avalanches, whose occurrence in time is not homogeneous (bottom).

**Figure 7.**
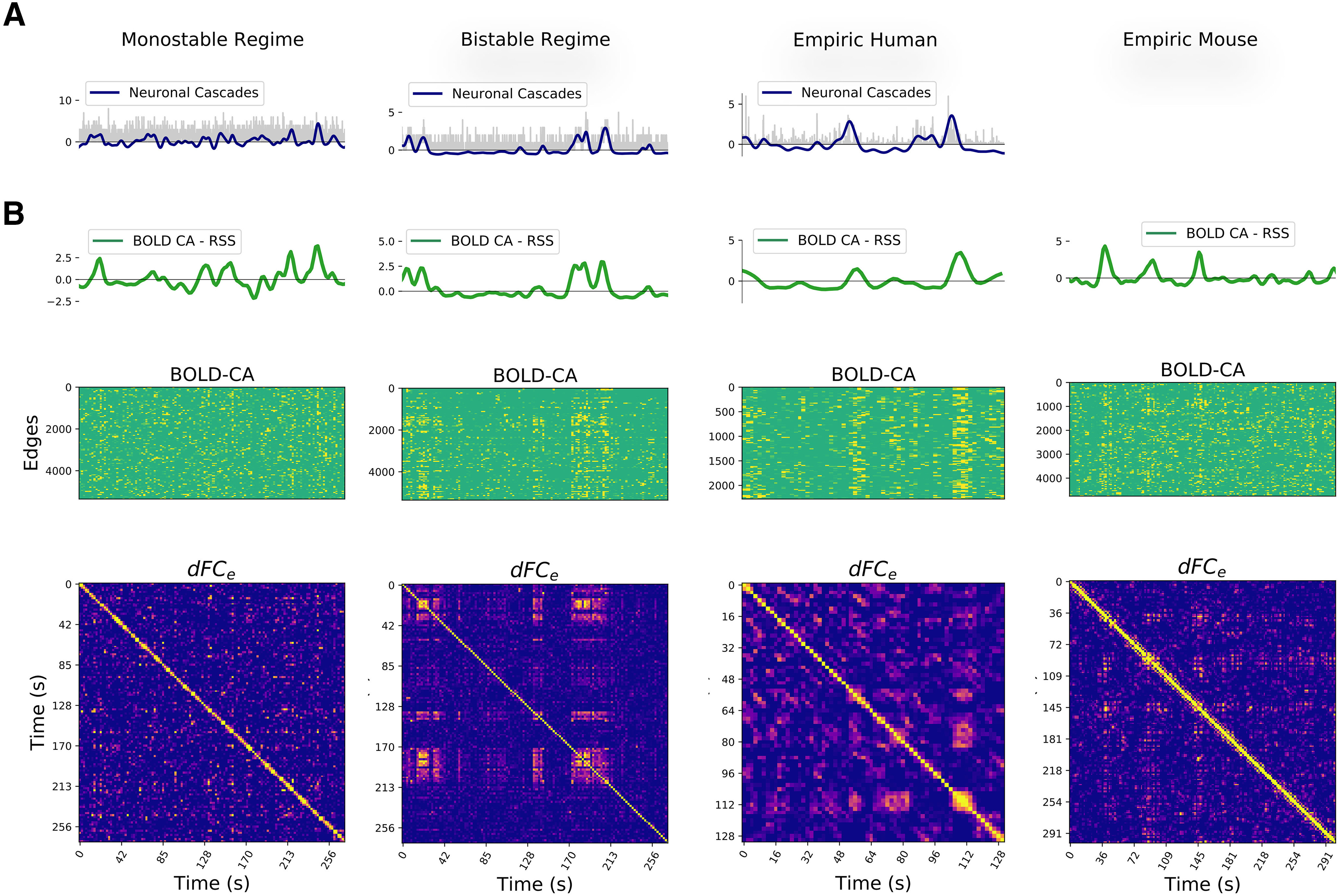
Neuronal cascades drive the functional dynamics. ***A***, Example of neuronal cascades in the monostable and bistable synthetic regime and for a representative subject of the empiric EEG/fMRI human dataset. ***B***, The BOLD-CA in simulated and empiric mouse and human datasets (middle panels) are characterized by sudden collective events involving large network parts (vertical stripes). The root sum square of BOLD-CA across all edges (RSS, green lines, top panels) defines the global CA amplitude signal for each dataset. Concurrently, the *dFC_e_* matrices (bottom panels) display both diagonal and off-diagonal blocks, remarking the non-trivial re-occurrence of the same stable functional network at distinct times (see Fig. 2*B*). At a visual inspection, the BOLD-CA events happen in coincidence with *dFC_e_* blocks and, most notably, the neuronal cascades and RSS signals (blue and green lines in panels ***B***, ***C***, respectively) co-fluctuate in most instances.

In the previous sections, we described how the large spontaneous deviations from baseline activity play a key role in the simulated system dynamics by activating specific large-scale functional networks. Here, we hypothesize that a qualitatively similar network behavior takes place in empiric resting-state data (see Discussion). To test this, we provide a working definition of neuronal cascades as a global measure of such long-lasting perturbations of the neuroelectric activity (see Discussion). For illustrating the pipeline, we use an example trial from an EEG/fMRI resting-state human dataset (see Materials and Methods). First, we binarize the firing rate activity in every brain region by assigning a unitary value when its activity exceeds ±3 σ (Fig. 6*A*). Then, we define the magnitude of the deviations from baseline by summing the binarized EEG over the regions (Fig. 6*B*, top, gray line). Finally, we convolve the obtained signal with a Gaussian kernel (width = 1.94 s = BOLD TR) and we downsample it to the same time resolution of the BOLD signals to allow comparison. The resulting neuronal cascade signal (Fig. 6*B*, top, blue line) is characterized by bumps which describe a long-lasting increase in the magnitude of non-standard perturbations (e.g., above 3 σ). Neuronal cascades should not be confused with the concept of neuronal avalanches (Beggs and Plenz, 2003), which consist of consecutive deviations from baseline activity of a subset of brain regions or localized neuronal groups (Fig. 6*A*, bottom, example of a neuronal avalanche in the red box). The number of regions in the subset defines the size of an avalanche, while the duration of the consecutive activations defines its lifetime (up to few hundreds of milliseconds in empiric data). Neuronal cascades are a global measure that describes a slower process on the order of tens of seconds. In Figure 6*B*, bottom panel, each red dot marks the occurrence of an avalanche of a certain size (dot size represents the duration). Remarkably, neuronal avalanches accumulate in correspondence with the largest neuronal cascades. Thus, we can think of the neuronal cascades as the clustering of neuronal avalanches. This first major result proves that the probability of observing strong brain fluctuations (including neuronal avalanches) is not constant in time, but increases during neuronal cascade peaks, which occur at an infra-slow time scale. Also, our simulation shows localized increases in cascade activity, which are shorter in the noisy monostable regime and more prolonged in the bistable regime (Fig. 7*A*).

**Figure 8.**
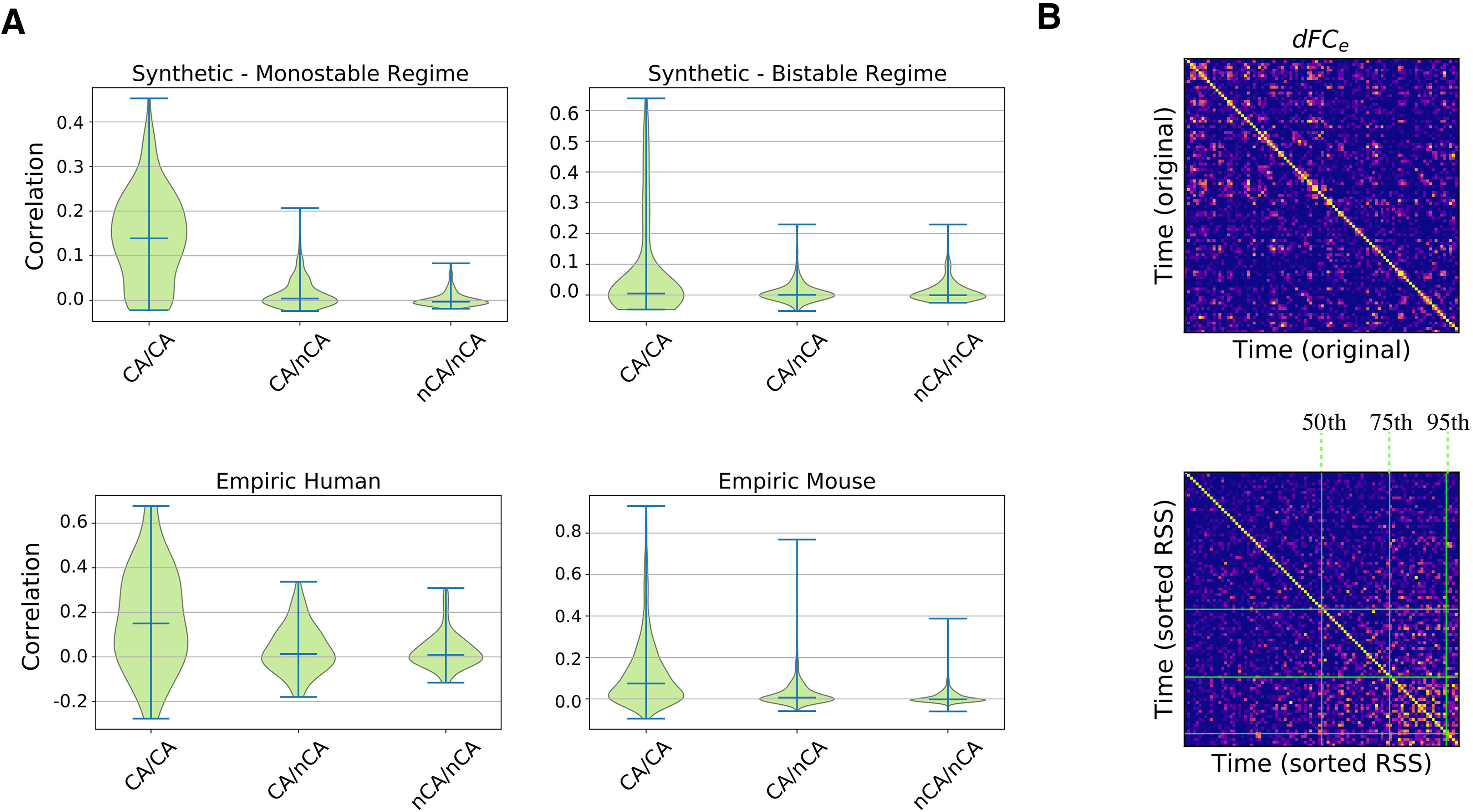
***A***, The largest BOLD-CAs events (CA, above the 98th percentile of the RSS; Fig. 4*C*, green line) are distinguished from non-events (nCA, below threshold). We report the synthetic and empiric correlations between *iFC*s at times within CA events (left in every panel), between CA events and non-events nCA (center of panels), within non-events nCA (right of panels). These correlations are by definition the off-diagonal values of the *dFC_e_* matrix (see Fig. 2*A*,*B*). The distribution of the correlations within events is wider and explains the greatest off-diagonal correlation values of the *dFC_e_* across all the synthetic and empiric datasets. This principle is explicitly shown in panel ***B***, where the original *dFC_e_* extracted from an empiric human trial (top) was sorted according to increasing RSS (bottom), leading to the clustering of high correlations toward high CA times. This shows that most of the non-trivial temporal correlations involve CA times falling in the last quartile of the RSS (above the 75th percentile, central green line). Thus, the strongest CA events drive the dynamics of *FC*.

At the level of fMRI, another *in silico* prediction is the presence of strong BOLD-CA events and their co-occurrence in coincidence with off-diagonal *dFC_e_* blocks. We verify the presence of BOLD-CA events in synthetic, empiric mouse fMRI and in human EEG/fMRI datasets (Fig. 7*B*, central panels), suggesting that temporally inhomogeneous bursts constitute a generic dynamic modality of real(istic) brain networks. To show the relation with *dFC_e_*, we first extract from the BOLD-CA signals the root sum squared (RSS) time series, which quantifies the amplitude of all edge CAs at each time point (Fig. 7*B*, green lines, top panels). Then, we partition the RSS signal in CA events and nCA non-events (respectively above and below the 98th percentile of the RSS values). We show that the correlation between any two events is statistically higher than the correlation between events and non-events as well as between couples of non-events (Fig. 8*A*). The results hold for synthetic and empiric results, which were pooled over several experimental trials. Since the correlation between CA patterns at different times correspond to the off-diagonal elements of the *dFC_e_* matrix (as shown in Fig. 2*A*,*B*), we conclude that bursts of BOLD-CA events account for the highest off-diagonal values in the *dFC_e_* in experimental datasets, as predicted by the model. In Figure 8*B*, we show an example of empiric human *dFC_e_* (top), and the same matrix where times are sorted accordingly to increasing RSS. Green lines represent different percentiles (50th, 75th, and 95th) of the RSS. Notice that, in line with the results above, the non-trivial temporal correlations (yellow off-diagonal *dFC_e_* elements) involve those time frames with the highest network CAs.

### Neuronal cascades subtend RSN dynamics

**Figure 9.**
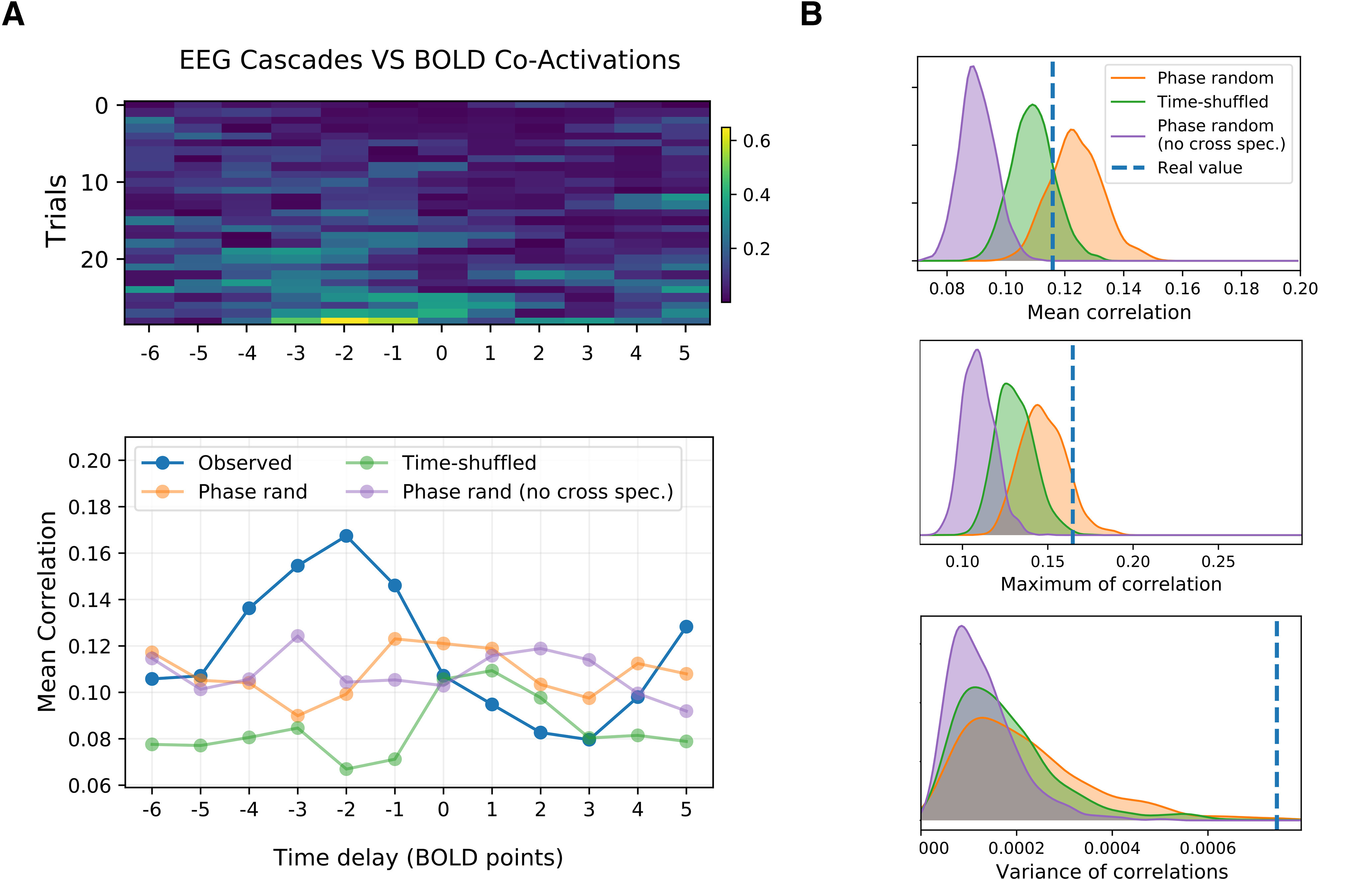
***A***, top, Correlation between the cascade magnitude and the BOLD-CA amplitude (Fig. 4*B*,*C*, blue and green lines) for different time lags in several EEG/fMRI trials extracted from a Human cohort of resting subjects. Negative lag is associated with a shift backward of the BOLD signal. ***A***, bottom, The cross-correlation averaged across trials shows a clear trend (blue line). The peak of correlation at lag –2 sampling points (1 pt = 1.94 s), as well as the rapid fall for positive lags, confirms that the EEG precedes the BOLD activity by few seconds. The same profile is evaluated by comparing the largest cascades with the BOLD-CA signals extracted from 1000 time-shuffled (example in green), 1000 phase-randomized (cross-spectrum preserved, example in orange), and 1000 phase-randomized (cross-spectrum not preserved, example in red) BOLD surrogates for every subject (see Extended Data Fig. 9-1*A*,*B* for surrogate properties). ***B***, The distribution of the mean, maximum, and variance of the cross-correlations for each surrogate model is displayed and compared with the empiric values. In particular, the variance plot shows a clear significance of the results (single subject results in Extended Data Fig. 9-1*C*).

Based on the theoretical results of the previous sections (summarized in Fig. 5), neuronal cascades should give rise to BOLD-CAs. In fact, a visual inspection of simulated and empiric data suggests the co-occurrence of neuronal cascades (blue lines in Fig. 7*A*) and BOLD-CA (RSS; Fig. 7*B*, green lines). Remarkably, a similar correspondence is present in an EEG/fMRI resting-state human dataset (best trial shown in Fig. 7), all the more since the simulations are done using a mouse connectome. To characterize this correspondence, we correlate the cascades signal with the BOLD-CAs amplitude profile. In both the monostable and the bistable regimes, the two measures are significantly correlated (*ρ* ∼ 0.54 and *ρ* ∼ 0.91, respectively). In Figure 9*A*, top, we report the correlations between the cascades and the BOLD-CA amplitudes across several trials of a human cohort (see Materials and Methods for trial selection). The correlation is conducted by shifting the RSS time series of a given time-lag. A peak of correlation appears naturally between the two measures when the BOLD signal is shifted backwards by two time points (3.88 s; Fig. 9*A*, bottom, blue line). Therefore, as expected, the EEG neuronal cascade signal precedes the BOLD-CAs by a few seconds. Notice that, in general, a shift forward of the BOLD activity results in a rapid loss of correlation.

10.1523/ENEURO.0283-21.2021.f9-1 Extended Data Figure 9-1 ***A***, Example of the edge CA time series in the original human dataset, compared to the respective phase-randomized and time-shuffled surrogates. Notice that the large CA events are preserved only in the first two surrogate models, but they are shifted in time. ***B***, Distribution of the BOLD-CA amplitudes (the RSS of the edge CAs) in the time-shuffled (same distribution as in the original dataset) and in the phase randomized surrogates where the cross-spectrum is preserved (orange) or not (purple). ***C***, Single-trial analysis of variance distribution (similar to Fig. 9*B*). The legend reports the p-values of the real observed variance versus the three surrogate distributions. Download Figure 9-1, EPS file.

Finally, to prove the statistical significance of our observations, we build surrogate BOLD signals and we compare the associated CA amplitudes to the neuronal cascade signal in each trial. In particular, we compare the observed cross-correlation profile (Fig. 9*A*, bottom, blue line) with the profiles extracted by correlation of neuronal cascades and surrogate CA amplitudes of three kinds: time-shuffled (example of cross-correlation profile in Fig. 9*A*, green line), phase-randomized, cross-spectrum preserved (example in Fig. 9*A*, orange line), and phase-randomized, cross-spectrum not preserved (example in Fig. 9*A*, purple line).

In all the surrogates, the functional dynamics is disrupted (see Materials and Methods). In the first two models, the static FC is preserved but its dynamic evolution is made trivial. Namely, the time-shuffled model consists of random *FC* jumps around a fixed pattern, while in the phase surrogate (cross-spectrum preserved), stationarity is strictly imposed by destroying any coherent fluctuation around the static *FC*. In fact, the latter corresponds to preserving the static correlations across node pairs, i.e., the average of the edge CA signals. These two surrogates also preserve the original fat-tail distribution of the BOLD-CA amplitudes (RSS values), and therefore supports bursty CA events (Extended Data Fig. 9-1*A*,*B*). When the cross-spectrum is not preserved, the static FC is corrupted and the CA amplitude distribution is normalized. For each surrogate type, we compute 1000 cross-correlation profiles. The distribution of their mean, maximum, and variance (averaged across all the trials) is shown in Figure 9*B*. The observed values are always significant when compared with the Phase-randomized surrogate with cross-spectrum not preserved. The observed mean values lie between the random (time-shuffled) and the ordered (phase-randomized) scenario (Battaglia et al., 2020). Only the Phase-randomized surrogate, where the cross-spectrum is preserved, reaches the peaks of correlation of the observed data, and the significance of the observed maximum is *p *=* *0.108. The variance of the correlation profile is always significant (*p* < 0.001) as compared with all the surrogate models. The result holds in several instances at the single-subject level (Extended Data Fig. 9-1*C*). We conclude that neuronal cascades drive BOLD-CAs dynamics thus shaping whole brain resting-state *FC*.

## Discussion

Cognitive function requires the coordination of neural activity across many scales, from neurons and circuits to large-scale networks (see Betzel and Bassett, 2017, and references within). As such, it is unlikely that an explanatory framework focused on any single scale will yield a comprehensive theory of brain activity, cognitive function or brain disease. Fast time scales evolve within the quasi-stationary constraints imposed by slow time scales. As the latter changes, the organization of the former must change consequently. Similarly, local neuronal events can trigger large-scale complex responses (Houweling and Brecht, 2008; Huber et al., 2008). We demonstrated this principle along the organization of the fast neuroelectric correlates of the slowly evolving large-scale *dFC* patterns for two mammalian species. Large neuronal cascades, defined by collective deviations of the simulated EEG signals away from baseline activity, can emerge as BOLD-CA events, i.e., strong simultaneous fluctuations at pairs of regions across the brain network. In turn, the intermittent set of BOLD-CA events underlies specific stable *FC* epochs. Thus, we suggest that large neuronal cascades have an organizational role in the resting-state brain dynamics.

In this work, we have adopted a theory-driven approach to reproduce qualitatively the resting state *FC*, analyze its dynamics and trace it back to its neuronal correlates. The NMM used to simulate regional dynamics maps the activity of an infinite number of all-to-all coupled neurons, each described by a simple phase-oscillator equation, into the mean-field firing rate and membrane potential variables (Montbrió et al., 2015). Although not giving full access to biophysical processes, as when using detailed single neuron models, the model we used preserves important information about the microscopic neural network organization of a simulated brain region (e.g., the neuronal synchrony; see Materials and Methods). The low computational load enables the simulation of fMRI-like sessions in TVB. Our results prove that *FC* fluctuations can be self-generated on a connectome by random sudden changes in the local neural network organization. In our setup, the analysis of more spatially fine grained neural dynamics, e.g., in the form of cell assemblies Buzsáki (2010), is not accessible. Future applications allow the possibility of including synaptic dynamics (Taher et al., 2020), adaptation (Gast et al., 2020), excitatory versus inhibitory populations (Montbrió et al., 2015; Laing, 2017; Dumont and Gutkin, 2019), among others. The use of these models establishes a new venue to address multiscale phenomena, maintaining, to a certain extent, the co-existence of microscopic and macroscopic scales of organization (Coombes and Byrne, 2019).

To simulate the spontaneous emergence of dynamic functional networks we tuned each neuronal population into a bistable regime. We expect similar results to hold for other models supporting local bistability. In our case, we can directly interpret the two stable states as two configurations of the spiking neural networks. Varying the global couplings, we discovered two regions of the parameter space, defining the monostable and the bistable regimes, where the large-scale organization is dynamical, but qualitatively different. In both regimes, the neuronal activity is characterized by up and down states, which occurrence was observed both *in vitro* (Plenz and Kitai, 1998; Cossart et al., 2003) and *in vivo* under several conditions such as anesthesia, slow-wave sleep, quiet waking and also during perceptual task across several species (Steriade et al., 1993; Luczak et al., 2007; Vyazovskiy et al., 2011; Engel et al., 2016; Jercog et al., 2017). The functional dynamics in the two regimes also differs, e.g., in terms of the statistical properties of the BOLD-CA events (Fig. 8*A*) or in the life-time of the stable *FC* states (i.e., the size of the *dFC* blocks in Fig. 7*B*, bottom).

The generation of a variety of dynamic regimes from changes in the global “environmental” parameters is in keeping with the degeneracy principle, according to which multiple models and/or parameter settings capture the neuronal variability (Marder and Taylor, 2011), and it is expected also in biological systems. For example, sleep deprivation, which alters brain and body function (Medic et al., 2017), is associated with changes in the speed of the *FC* evolution (Lombardo et al., 2020). Accordingly, distinct features of the *dFC* should correspond to a different underlying neuroelectric organization (and perhaps to distinct mechanisms of communication across brain regions; Battaglia and Brovelli, 2020). The transition between wakefulness and sleep brain states offers an example of sudden change of the whole-brain dynamics associated with the microscopic neuroelectric re-organization (Larson-Prior et al., 2009; Boly et al., 2012; Tagliazucchi et al., 2016; El-Baba et al., 2019).

While the debate over the actual nature of *FC* dynamics is still ongoing (Hindriks et al., 2016; Laumann et al., 2017; Liégeois et al., 2017), several evidences were provided in favor of the *dFC* as a legitimate measure of the functional evolution (Preti et al., 2017). Here, we found that a large part of the functional information is condensed in large BOLD-CA events, in keeping with previous results (Zamani Esfahlani et al., 2020; see Fig. 5*A*). The occurrence of the BOLD-CA events cannot be ascribed to the spectral properties of the BOLD signals nor to motion artifacts (Zamani Esfahlani et al., 2020), which supports a genuine phenomenon. Our results provide an *in silico* support to a burst-based system dynamics where correlated CA events subtending RSNs occur intermittently and are separated by long low-activity periods. These results are in accordance with previous works showing that the relevant information about the major RSNs is carried by the largest fluctuations of the fMRI BOLD signals (Tagliazucchi et al., 2012a; Liu and Duyn, 2013; Allan et al., 2015; Cifre et al., 2017; Gutierrez-Barragan et al., 2019). The spontaneous bursts of BOLD-CA highlight recurring sets of network edges, which subtend special RSNs. How the system is organized around these preferential sets and what triggers the sudden co-fluctuations remain to be determined. Our analysis remarks the central role of the structural connectivity in this process, in line with a recent work suggesting that CA are shaped by structural modules (Pope et al., 2021). Here, we suggest that large neuronal cascades generated by sudden neuronal re-organization in source regions may play a central role in driving the exploration of the connectome and in determining the large-scale *FC* dynamics.

During the BOLD-CA events, the evolution of the *FC* slows down, as shown by the high correlation between functional states at consecutive times (Fig. 7*B*, bottom panels). An emergent stable *FC* elicited by a BOLD-CA event can persist for several seconds. The same state can re-appear after a few minutes in coincidence with another CA. In many dynamical systems, slowdowns and large-scale events are typically observed at the transition between different states (or phases), i.e., at the critical point (Scheffer et al., 2009). Critical dynamic systems are characterized by other typical properties, including the presence of fluctuations at all spatiotemporal scales (Cocchi et al., 2017). Historically, the hallmark of a critical organization of the brain activity came from the observation of neuronal avalanches whose size and duration fit a power-law (Beggs and Plenz, 2003; for a “critical” look at brain criticality see Beggs and Timme, 2012).

In order to compare the fast EEG and the slower BOLD signals, we focused on the slow unfolding of the EEG deviations from baseline activity. In other words, instead of focusing on the size and duration of the neuronal avalanches, we analyzed their collective occurrence at the resolution of seconds, which we refer to as neuronal cascades (Fig. 6). We showed that the cascade magnitude is correlated to the BOLD-CA amplitude (Fig. 9*A*). In particular, in the empiric human dataset we observe a rapid drop of this correlation when the BOLD activity is shifted backward with respect to the EEG signal. This fact makes our result more robust since we expect a change of the BOLD activity to happen after a sustained neuroelectric activity.

Notice that in the empiric data set, not all the neuronal cascades give rise to BOLD-CAs, and not all the BOLD-CAs are preceded by large neuronal cascades. In general, we should expect a non-trivial interaction between these phenomena and other infraslow processes. For example, a growing corpus of evidence relates the slow functional and neuroelectric dynamics with other physiological and cognitive infraslow processes such as neuromodulation (Shine et al., 2016), visceral (heart and gut) inputs (Azzalini et al., 2019), and cognitive performance (Monto et al., 2008).

In the cases in which neuronal cascades and BOLD-CA events co-occur, we can hypothesize that the stable *FC* state evoked by a CA event is related to the specific structural channels in which the neuronal cascade spreads. In fact, we showed in the simulated data that the largest neuronal cascades underlie specific functional patterns (Fig. 5), depending on the location of the onset. Also, the observation that the same cascade appears at regular intervals (see for example the three peaks at second ∼100, ∼200, and ∼300 in Fig. 5*A*) suggests that the re-occurrence of the same stable patterns in time can be traced back to the increased probability of the same cascade to occur following a first large event, in the same way an after-shock follows the main earthquake. It is interesting to note that, as in our model, correlated bursty events associated with memory effects are observed in many natural systems (Karsai et al., 2012).

The above hypotheses require further exploration of larger empirical datasets. In the model, we have shown that the multiscale mechanistic origin of large cascades can be understood ultimately by the interplay between the local neuronal organization, the stochastic nature of its sudden re-organization and the structural constraints it obeys. The present work provides a new understanding of whole-brain functional dynamics, which is shaped by neuronal cascades giving rise to large BOLD-CA. Since most neurologic disorders are characterized by complex reorganizations at the neuronal scale, it will be interesting to determine whether specific alterations in neuronal cascades sign specific neurologic disorders, and explain the already identified alterations in whole-brain dynamics.

## Synthesis

Reviewing Editor: Gustavo Deco, Universitat Pompeu Fabra Decisions are customarily a result of the Reviewing Editor and the peer reviewers coming together and discussing their recommendations until a consensus is reached. When revisions are invited, a fact-based synthesis statement explaining their decision and outlining what is needed to prepare a revision will be listed below. The following reviewer(s) agreed to reveal their identity: Anders Lansner.

Reviewer 1:

This paper attempts to link local and global brain dynamics to predict neural activity, EEG, and BOLD, with particular focus on large excursions in BOLD that it terms “switching” behavior.

Unfortunately the paper has very serious problems with writing, model formulation, analysis, and conclusions. I can’t recommend it for publication in anything like its present form.

The following comments are not exhaustive, but indicate the main areas of difficulty.

Writing

1. Many terms and phenomena are introduced without precise definition, or insufficient definition for an interdisciplinary audience. Examples: nullcline, resting state network, phase plane, dynamical element, avatar, motif, hubness, basin of attraction, homeostatic agents, nonstandard perturbation, degeneracy principle, a bursts-based system dynamics, uniform unfold, etc., etc., etc. In some cases, it is possible to work out what is meant, in others these things remain ambiguous even for someone who works in this area - authors must define their terms.

2. Other concepts are treated as if they are accepted fact when they are not. These need discussion and justification. Examples: switching behavior of FC.

3. Figures are extremely poorly explained. Captions don’t refer to all the elements of each frame. Much of the text in captions actually belongs as part of the argument in the main text to avoid the need to keep jumping back and forth. Captions should describe the linestyles, color bars, axes, and other elements, while the discussion belongs in the main argument in the text.

4. Many features of figures are left for the reader to work out - both what they are and what their relevance to the argument might be. All figure elements not used explicitly in the argument should be deleted - figures are not decorations and should only be in the paper if they are directly supporting the argument.

5. Many figures are too small to read. Constantly having to zoom in and out in the electronic version is a sign that too much has been packed in and insufficient thought has been given to what are the essential elements. Requiring that all figures be readable in a normal-sized printout and redesigning them accordingly would be a useful criterion to ensure that the key points are conveyed clearly and that “decorative” and unnecessary aspects are deleted.

6. Sections should normally begin with a brief preamble so the reader has some preview of what kind of things they contain.

7. There is inadequate acknowledgment of the vast literature on multiscale brain dynamics, studied by various approaches including close relatives of this one.

Modeling and Analysis

1. The model is not properly justified and appears to have been assembled from near-random ingredients. Why QIF neurons? Why a discrete connectome of a particular coarseness? Why only excitatory connections betwen nodes? Why are nodes treated as real entities, when they are merely artifacts of the particular discretization used? How big is the noise in each case discussed?

2. It is stated that V is a potential, I is a current, etc. If that is so then Eq. (1) and everything that follows is definitely wrong - the terms have different units and dimensions and can’t be summed. Later, velocities are inserted in physical units, but that is unlikely to be compatible with nondimensionalized units elsewhere. I suspect that various nondimensionalizations have been done, but none of these are stated, so it is hard to be sure what parts are mutually contradictory and what parts might be made compatible.

3. The connectivity is only excitatory, but white matter connections between cortical areas impinge on both excitatory and inhibitory neurons and the latter can, at times, be more greatly excited. In any event, inhibition is extremely important in the attainment of near-criticality, which is important according to the Abstract. How then can the neglect of inhibition be justified. As purely a toy model of the dynamics on a toy model of the connectivity this might be fine, but it is not a model of the brain and no serious conclusions about brain dynamics can be drawn from it. It might be publishable, with major rewriting in an abstract network dynamics journal.

4. No tests of the sensitivity of the results to the particular artificial discretization have been done. Indeed, some of the analyses - e.g., via motifs and hubness - treat the discretized network as being the real object of study. Motif topology, for example, will be changed if the discretization is made finer and nodes are subdivided - e.g., closed triangles will no longer be closed. What then is the justification for such analyses?

5. No serious justification is given of the various types of FC and what the advantages/disadvantages of each are. Likewise, the various analyses are introduced ad hoc without explaining (in many cases) why each is the appropriate method for the experimental data and numerical outputs at hand. For example, how does computation of a hybrid dFC allow one to “check the correspondence” of two other matrices.

6. How does the flipping between states relate to stochastic resonance?

7. Is there any evidence that large areas of the brain really flip between widely different low and high firing-rate states? Wouldn’t the latter correspond to some kind of seizure? What firing rates are predicted and are these realistic? What EEG spectrum is predicted and does it accord with experiment? These questions will need the results to be converted from nondimensional units to dimensional ones.

8. line 96 - do the authors seriously believe that there are “poorly connected” regions of the brain? Histology shows that there are connections everywhere. It is necessary to rule out (or at least acknowledge) possibilities such as white matter connections being underestimated in the measurements, fewer but stronger connections being present, etc. Structural connectivity can differ widely from effective connectivity.

9. line 123. What kind of sliding window is used? Is it tapered?

10. line 143. Justify the use of average firing rate as being EEG; also the connection to BOLD. This is just one of several points where unjustified “bridges” to real data appear in the argument. Later (see below) these are used to make overblown claims about the correspondence to actual brain dynamics.

11. line 178: “variance” of elements at fixed time, all elements across time, or what?

12. line 313: explain the role of the first leading eigenvector;

13. There are many places where the only analysis is to say that the outcomes are “similar", “give the impression", “seem to account", etc. This is scarcely adequate in a refereed paper. Speculative remarks should be clearly demarcated as such.

14. It has been shown in the literature that much of the appearance of off-diagonal block-like structure in connection matrices is actually an artifact of the mapping of higher-dimensional physical structure to a one-dimensional list of nodes. To prove the existence of blocks, visual inspection of the matrices is not sufficient - one would need to do a proper community-detection analysis.

15. There has been no assessment (or acknowledgment) of the fact that large numbers of parameters have been adjusted to get rough qualitative agreement with dynamics. What is the choice? How robust is it? Is the outcome just from fine tuning by hand? If other phenomena are not reproduced, how does one justify the parameter choice?

Discussion/Conclusions

1. Given the above problems, the conclusions drawn are not justified - especially that the results are “biologically realistic”. At best, the dynamics found are vaguely reminiscent of observations.

2. The section proceeds largely via various nebulous arguments with semi-defined terms that give the impression of a connection between what has been done and real brain dynamics. I found this quite misleading. For example “slowdowns", “accelerations", “speeds", “absorption times", are all ambiguous. Vagueness like “might be", “preserving important [but undefined] information", “we suggest", intrudes at many points. Many of the statements are true as far as they go, but have little content and say little or nothing about actual brain dynamics.

3. The connection to EEG and BOLD measurements is asserted but not established. To make such a connection, dimensional results are needed that can be quantitatively compared. It would be possible to formulate a host of two-state local dynamical models, place them at nodes of some kind of connectome, and confidently expect that cascades of dynamical changes would be seen - indeed universality in statistical mechanics says that an infinity of such models should exist (e.g., as studied in self-organized criticality). Why this one? The difficulty is not to come up with models, but to choose a model based on tight biological constraints that rule out almost all alternatives (including, most probably, the present one).

Many more comments along the above lines could be made. The most important point is that the field has progressed and it is no longer appropriate to build toy models and assert that they have direct biological relevance. TVB makes it very easy to do this, but not every combination that can be run on that platform (indeed almost none, given the combinatorial possibilities) is sensible. Toy models have their place, but need to be acknowledged as such and published in an appropriate journal without claims that they directly explain data. Moreover, as described above, this particular toy model has many problems in its formulation and analysis that would need to be fixed before it could be published anywhere.

Reviewer 2:

Review report on manuscript eN-NWR-0283-21, entitled “Neuronal cascades shape whole-brain functional dynamics at rest"

General summary

This manuscript describes a reduced simulation model of many connected cortical areas and results demonstrate the dynamical features displayed which are compared with simultaneous BOLD and EEG data from human and mouse. The model itself is developed in The Virtual Brain simulation environment and represents each cortical area with one non-spiking computational unit with average membrane potential (vm) and spiking rate (r). These neural units were derived by others as the thermodynamic limit of an infinite number of quadratic IF model neurons. The connectivity of the network with regard to existence and strength of connections is setup from connectomics data obtained from Allen brain atlas on mouse and from structural connectivity data from MRI in humans.

The results reported describe the dynamic behavior of Vm, R, and synthetic BOLD and EEG (from Vm) as well as their co-fluctuations. The model displays slow fluctuations with down and up states and co-occuring such states between well connected area nodes. Also, e.g. BOLD and EEG are co-fluctuating and in certain regions of parameter space also display what they call neuronal cascades, which co-occur in the different measures and are concluded to be the major dynamic processes behind spontaneous fluctuations in the brain’s resting state.

Major comments

The manuscript is overall well written, clear and interesting to read. The results are validated and discussed with a critical view and possible extensions to the model, like adding adaptation and fast synaptic plasticity, are suggested and welcome in future works. While reading, several questions come up that should be brought up and discussed in the revised version:

(1) The computational model is very coarse, but is still stated to connect micro and macro scales of brain dynamics (line 473). I think this is overstated, because most likely, the many millions of neurons in a cortical area are probably not showing qualitatively the same dynamics, but are most likely partitioned in subgroups like, for instance, cell assemblies that represent part of some “mental object” and are connected to other brain areas as well. The resting state called “Default Mode Network” is believed to reflect spontaneous activity supporting memory and other “inner” cognitive processes. Real neuron firing rates have a distribution from 0 to several tens of Hz, and data from cortical recordings (e.g. Quiroga 2012) suggest that activity in higher order human cortex is very sparse, with only a fraction of principal neurons being coding with higher spike rates, while the majority are silent. It should be discussed if and in what way the spiking rates in the range 0-2 Hz and dynamics of the proposed coarse grained model is compatible with such a mesoscopic view of cortical dynamics.

(2) The more complex model dynamics is entirely noise driven, but real pyramidal cells and also some inhibitory interneurons have a rather prominent adaptation and also depressing/facilitating synapses, which are main player in cortical models (Gast el al. 2020, Fiebig and Lansner 2017). I think some reservations on the model fidelity should be added in the discussion, in the section where it is stated that such features could be added to the model in the future.

(3) Weights between the model’s area nodes derived from connectomics data are always positive. For sure, long and cortico-cortical axons are from excitatory pyramidal cells, but they are also terminating on local inhibitory interneurons, which invert their sign (e.g. D’Souza and Burkhalter, 2017). It has been suggested that top-down inter-area connectivity is predominantly inhibitory (Zhang et al. 2014). Net negative weights might introduce competition in the system and also prevent runaway excitation. Does the latter occur in the current model or what prevents it from occuring - I fail to understand that. Thess matters should be brought up and discussed in the Discussion.

(4) For the mouse, where the connectomics data was used for the model, are the subgroups of co-activating areas in any way similar to the subgroups in the experimental data? Fig. 3 show only minor similarity between model and experiment. Why is that?

Minor comments

(1) Though the mean field model is theoretically derived, which is a bonus. It has two key state variables, vm and r, which take on values in the ranges [-2,1.5] V? and [0,2] Hz respectively. The NMM has parameters Vp and Vr the values of which I did not find in the manuscript. They should be added.

(2) The term “standardized firing rate” is used on line 299 and displayed in Fig. 2b but not defined. Should be defined.

(3) Line 155: should be “... as the BOLD"

(4) The term “brain avatar” is used in several places about the model? Is that something else than a “brain model", please explain the relevant difference or avoid introducing new terminology.

(5) Line 351: “... in the monostable <what?> ...”

(6) Line 397: “... in most instances"

(7) Line 399-400: Incomplete sentence.

(8) Line 478: “... full access to ...”, I suggest “... full justice to ...”

(9) Line 561: remove one “be"

References

RQ Quiroga (2012) Concept cells: the building blocks of declarative memory functions,

Nature Reviews Neuroscience 13 (8), 587-597;

R Gast, Schmidt H, Knösche TR; A Mean-Field Description of Bursting Dynamics in Spiking Neural Networks with Short-Term Adaptation (2020). Neural Comput 32 (9): 1615-1634. doi: https://doi-org.focus.lib.kth.se/10.1162/neco_a_01300;

F Fiebig and Lansner A (2017). A Spiking Working Memory Model Based on Hebbian Short-Term Potentiation, J Neurosci 37: 83-96. https://doi.org/DOI: http://dx.doi.org/10.1523/JNEUROSCI.1989-16.2016;

RD D’Souza., Burkhalter A (2017). A Laminar Organization for Selective Cortico-Cortical Communication, Frontiers in Neuroanatomy 11:71, DOI=10.3389/fnana.2017.00071;

S Zhang, Xu, M., Kamigaki, T., Hoang Do, J. P., Chang, W. C., Jenvay, S., Miyamichi, K., Luo, L., & Dan, Y. (2014). Selective attention. Long-range and local circuits for top-down modulation of visual cortex processing. Science 345: 660-665. https://doi.org/10.1126/science.1254126.

## Author Response

Letter to the reviewers

Review Synthesis:

General comments on the revision

I think the manuscript has been improved by the revision and it was interesting and informative to read for me as a non-expert on this particular dynamics and neural mass models.

We appreciate the comments provided by the reviewers. We have addressed all the remaining comments in the revision. We provide a pdf with the revised version of the paper with all changes in red.

Major comments

I have a few remaining concerns:

1) (see also (1) in my original review) The connection between micro and macro scales is still problematic and overstated. Regarding temporal scales perhaps, but the spatial scales are definitely not bridged by this model. It is good enough to say that this slowly fluctuating resting state provides a background on which more spatially fine grained neural dynamics in the form of e.g. cell assemblies can superimpose. But the current model says nothing about the nature of these micro scale dynamics, which should be made clear.

We tuned down the reference to the microscopic scale throughout the text (in red). In fact, as the reviewer suggested, this might be misleading. In the discussion we write

“…Our results prove that functional connectivity fluctuations can be self-generated on a connectome by random sudden changes in the local neural network organization. In our setup, the analysis of more spatially fine grained neural dynamics e.g., in the form of cell assemblies, is not accessible.

Future applications allow the possibility…”

2) As far as I understand, the model derived from connectomic data shows neural cascades and dFC involving similar constellations of areas as in empirical data. If this is the case, it is an important result that should be better described and communicated.

In this work, the parameters of the model were tuned to show a realistic temporal dynamics i.e., a non-trivial dFC which is associated with bursts of BOLD co-activations. These bursts highlights specific functional networks (Fig.5). However, we did not tune the parameters to spatially fit the empirically known resting state networks, since it was not our purpose here. Therefore, while the functional connectivity is spatially structured, this structure is not compared to empiric data. We agree that this is an important point and we are working on both spatial and temporal characterisation of resting state networks in a followup work.

Minor comments

1) Line 2: What is a “recurrent brain state”?? I know of recurring states and recurrent connectivity but not this. Please clarify.

To be more clear, we changed it with “recurrent functional connectivity states”

2) Lines 40 and 597 “... keeps track off” is not a very precise wording. A model "reproduces", for instance.

We substitute by

..Dynamic FC “measures” FC fluctuations..

and

..Differently from standard NMMs, the chosen model “quantifies” the internal amount of synchronization ..

3) Line 117: “..., which detailed connectome is imported ... , suggest “..., the detailed connectivity of which was imported…”

We substituted it by “the detailed connectivity of which was imported”

4) Line 544: “The empirical human EEG/fMRI ..:”

We corrected it

We thank the reviewers for their valuable suggestions.
